# Attachment of the RNA degradosome to the bacterial inner cytoplasmic membrane prevents wasteful degradation of rRNA in ribosome assembly intermediates

**DOI:** 10.1371/journal.pbio.3001942

**Published:** 2023-01-05

**Authors:** Lydia Hadjeras, Marie Bouvier, Isabelle Canal, Leonora Poljak, Quentin Morin-Ogier, Carine Froment, Odile Burlet-Schlitz, Lina Hamouche, Laurence Girbal, Muriel Cocaign-Bousquet, Agamemnon J. Carpousis

**Affiliations:** 1 LMGM, Université de Toulouse, CNRS, UPS, CBI, Toulouse, France; 2 IPBS, Université de Toulouse, CNRS, UPS, Toulouse, France; 3 Infrastructure Nationale de Protéomique, ProFI, Toulouse, France; 4 TBI, Université de Toulouse, CNRS, INRAE, INSA, Toulouse, France; HHMI, Massachusetts Institute of Technology, UNITED STATES

## Abstract

RNA processing and degradation shape the transcriptome by generating stable molecules that are necessary for translation (rRNA and tRNA) and by facilitating the turnover of mRNA, which is necessary for the posttranscriptional control of gene expression. In bacteria and the plant chloroplast, RNA degradosomes are multienzyme complexes that process and degrade RNA. In many bacterial species, the endoribonuclease RNase E is the central component of the RNA degradosome. RNase E-based RNA degradosomes are inner membrane proteins in a large family of gram-negative bacteria (β- and γ-Proteobacteria). Until now, the reason for membrane localization was not understood. Here, we show that a mutant strain of *Escherichia coli*, in which the RNA degradosome is localized to the interior of the cell, has high levels of 20S and 40S particles that are defective intermediates in ribosome assembly. These particles have aberrant protein composition and contain rRNA precursors that have been cleaved by RNase E. After RNase E cleavage, rRNA fragments are degraded to nucleotides by exoribonucleases. In vitro, rRNA in intact ribosomes is resistant to RNase E cleavage, whereas protein-free rRNA is readily degraded. We conclude that RNA degradosomes in the nucleoid of the mutant strain interfere with cotranscriptional ribosome assembly. We propose that membrane-attached RNA degradosomes in wild-type cells control the quality of ribosome assembly after intermediates are released from the nucleoid. That is, the compact structure of mature ribosomes protects rRNA against cleavage by RNase E. Turnover of a proportion of intermediates in ribosome assembly explains slow growth of the mutant strain. Competition between mRNA and rRNA degradation could be the cause of slower mRNA degradation in the mutant strain. We conclude that attachment of the RNA degradosome to the bacterial inner cytoplasmic membrane prevents wasteful degradation of rRNA precursors, thus explaining the reason for conservation of membrane-attached RNA degradosomes throughout the β- and γ-Proteobacteria.

## Abstract 2

This study shows that the physical separation of early steps in ribosome assembly in the Escherichia coli nucleoid from the RNA degradosome attached to the inner cytoplasmic membrane protects precursor ribosomal RNA in immature particles from degradation.

## Introduction

In *Escherichia coli*, ribonuclease E (RNase E) has a central role in the processing of stable RNA (rRNA and tRNA) and in the degradation of mRNA. RNase E homologues are widely distributed in bacteria and the plant chloroplast [1,2]. The N-terminal half of *E*. *coli* RNase E has a compact structure that is the site of endoribonuclease activity, whereas the C-terminal noncatalytic region is predominantly natively unstructured protein [3–5]. The noncatalytic region has small motifs (15 to 40 residues) known as microdomains or Short Linear Motifs (SLiMs) that serve as sites of interaction with proteins, RNA, and phospholipid bilayers [1,2,5,6]. In *E*. *coli*, the exoribonuclease PNPase, the glycolytic enzyme enolase, and the DEAD-box RNA helicase B (RhlB) bind to RNase E microdomains to form the multienzyme RNA degradosome [7–11]. Another microdomain, known as the Membrane-Targeting Sequence (MTS), forms a 15-residue amphipathic alpha-helix that attaches RNase E to the phospholipid bilayer of the bacterial inner cytoplasmic membrane [6,12]. Protein sequence comparisons have shown that RNase E homologues in the β- and γ-Proteobacteria have a conserved N-terminal catalytic domain and a large natively unstructured C-terminal half with microdomains including an MTS [1]. Thus, throughout these phyla of bacteria, RNA degradosomes are predicted to be membrane-attached.

Fluorescence microscopy of live *E*. *coli* cells has shown that RNase E is localized to the periphery with no detectable protein at the center of the cell where the nucleoid is located [6,12,13]. Systematic analyses of the inner membrane proteome have confirmed that RNase E is an inner membrane protein [14,15]. RNase E forms small clusters (puncta) on the inner cytoplasmic membrane [12,16]. RhlB and PNPase colocalize with RNase E, thus confirming association with these enzymes in live cells [16]. Inhibition of transcription by rifampicin, which depletes mRNA as well as precursors of rRNA and tRNA, results in the disappearance of puncta, thus suggesting that RNA substrate is required for RNA degradosome clustering [12]. However, recent work has shown that kasugamycin, which inhibits the initiation of translation, also results in disappearance of RNA degradosome puncta [16,17]. Since the synthesis of mRNA, rRNA, and tRNA continues in the presence of kasugamycin, it has been proposed that polyribosomes are necessary for RNA degradosome clustering. This experimental work suggests that inner membrane puncta are sites of mRNA degradation in which the initial step involves capture of polyribosomes by the RNA degradosome.

Recent work in *E*. *coli* has shown that the RNA degradosome can be displaced from the inner cytoplasmic membrane under conditions of stress. Upon transition from aerobic to anaerobic growth, cells filament and RNase E localizes to the interior of the cell in a diffuse pattern [18]. Starvation for a nitrogen source results in the formation of a single large RNase E focus [19]. Treatment of cells with the protein synthesis inhibitor chloramphenicol results in the formation of RNase E foci that are not attached to the inner cytoplasmic membrane [16]. These observations suggest that stress-induced detachment of RNase E from the inner membrane could control RNase E activity or accessibility to RNA substrates. The mechanism that triggers detachment of the RNA degradosome from the bacterial inner cytoplasmic membrane remains to be elucidated.

Although RNase E is an essential enzyme in *E*. *coli*, mutant strains encoding variants in which part or all of the C-terminal noncatalytic region is deleted are viable [10,20–22]. Binding to the bacterial inner cytoplasmic membrane is disrupted in mutant strains in which the amphipathic α-helix formed by the MTS is disrupted by amino acid substitution or deletion [6]. In an *rne(ΔMTS)* mutant strain, the *rne* gene encodes a variant in which the MTS has been deleted [12,13]. This variant is distributed uniformly throughout the cell. For clarity, we will refer to the wild-type enzyme as inner-membrane-RNase E (imRNase E) and the variant as nucleo-cytoplasmic-RNase E (ncRNase E). In the mutant strain, growth and mRNA degradation are slower than in the isogenic wild-type control strain [23]. Although a previous study suggested that membrane localization of RNase E preferentially destabilizes mRNA encoding inner membrane proteins [13], this preference was not considered significant in a subsequent study, which concluded that the slowdown of mRNA degradation in the *rne(ΔMTS)* mutant strain is global [23].

Evidence accumulating over the past 2 decades suggests that ribosome assembly and rRNA processing are spatially organized [24]. In *E*. *coli*, despite their separation by hundreds of kilobase pairs of DNA on the bacterial chromosome, most rRNA operons are in close proximity, suggesting that there is a bacterial nucleolus [25]. Ribosome assembly is a complex process that requires the coordinated synthesis of rRNA and ribosomal proteins (r-proteins) [26,27]. Ribosomal RNA is transcribed as a 30S precursor, which then undergoes extensive processing carried out by a battery of ribonucleases including RNase III and RNase E [26,28–30]. Imaging of chromosome spreads has shown that r-proteins bind cotranscriptionally to nascent rRNA [31]. Recent work has elucidated the structure of an rRNA transcription elongation complex that promotes cotranscriptional rRNA folding and r-protein binding [32]. Some maturation steps, such as processing by RNase III, take place on nascent rRNA in the bacterial nucleoid [31,33]. In addition to r-protein binding, there are many ribosome assembly factors, including rRNA modification enzymes, RNA helicases, and protein chaperons. Kinetic analyses have identified 2 intermediates in the 30S assembly (p_1_30S and p_2_30S) and 3 intermediates in the 50S assembly (p_1_50S, p_2_50S, and p_3_50S) [34]. However, recent work employing quantitative mass spectrometry and single-particle cryoEM has shown that ribosome assembly pathways are more complex than previously believed [26,27,35]. Ribosome assembly involves cooperative rRNA folding blocks that correspond to structural domains in mature 30S and 50S ribosomal subunits. Therefore, multiple parallel assembly pathways result in a heterogeneous population of intermediates.

Considering the complexity of the ribosome assembly process, it is not surprising that occasional errors result in dead-end intermediates, which are then removed by a quality control process. Recent work has shown that degradation of rRNA in ribosomal subunits is initiated by RNase E cleavage [36–39]. Here, we show that ncRNase E in the bacterial nucleoid interferes with normal ribosome assembly resulting in wasteful degradation of newly synthesized rRNA. We suggest that the slowdown in mRNA degradation in the *rne(ΔMTS)* mutant strain is an indirect effect involving the degradation of rRNA in ribosome assembly intermediates. In the wild-type bacterial cell, we propose that degradation of dead-end intermediates in ribosome assembly is initiated by imRNase E after they are released from the bacterial nucleoid. We compare and contrast our results to recently published work with another family of gram-negative bacteria (α-Proteobacteria) in which RNase E-based RNA degradosomes are localized to ribonucleoprotein granules in the interior of the cell.

## Results

### A genetic link between RNase E localization and ribosome assembly

**Fig 1:**
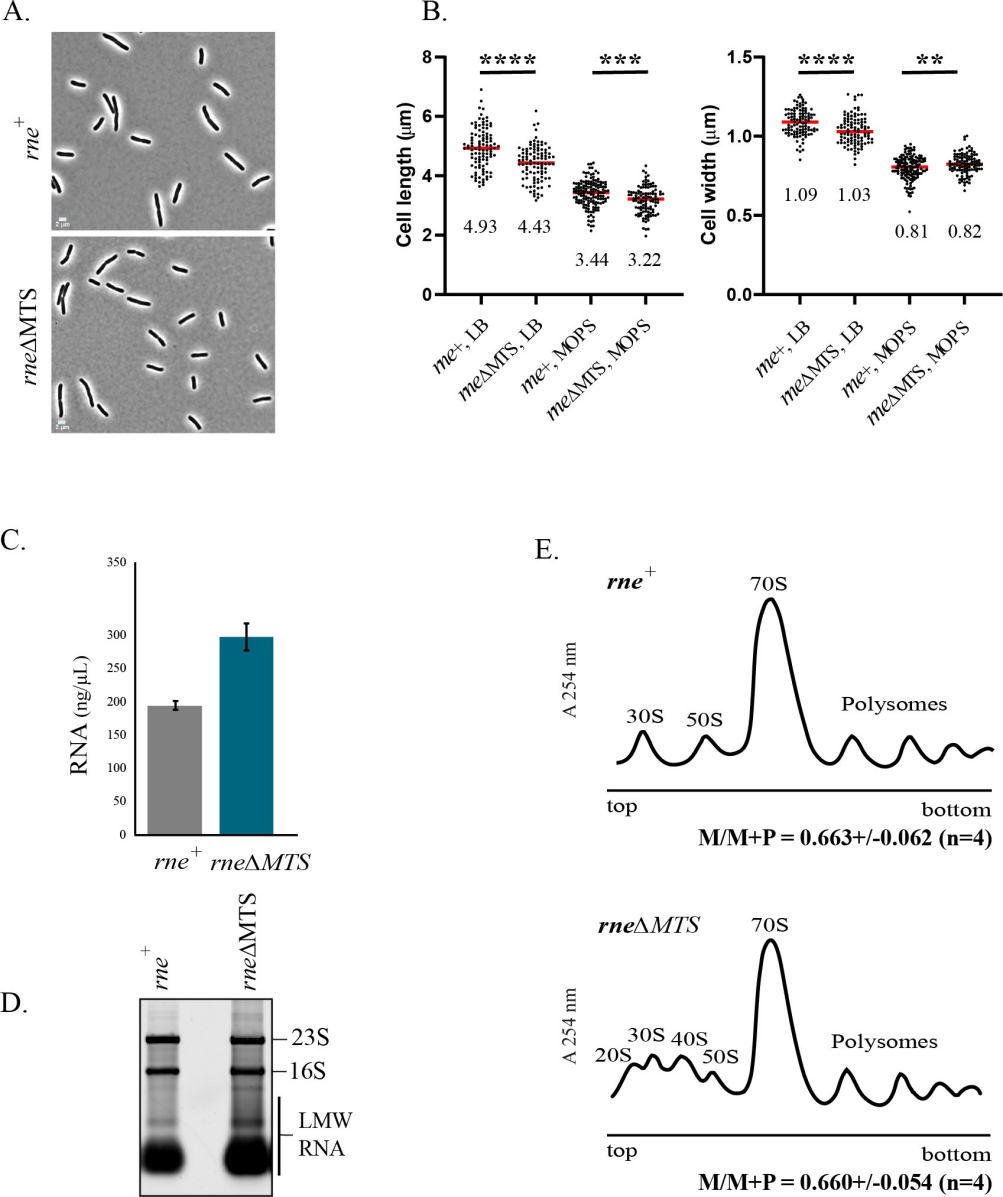
Cell size, RNA content, and polysome profiles. **(A)** Phase-contrast images. Micrographs of strains expressing either membrane-bound (*rne*^+^) or cytoplasmic (*rne*ΔMTS) RNase E were made at the same magnification. **(B)** Cell size. Lengths and widths were measured as described [39]. Scatter plots showing median cell length and width of *rne*^+^ and *rne*ΔMTS strains grown in either LB or MOPS media. Cells from 2 independent experiments (*n* > 100) were analyzed by ImageJ using the MicrobeJ plugin. Median length and widths (μm) are shown below each plot. *P* values were calculated using a parametric unpaired *t* test (GraphPad Prism): **** = *P* < 0.0001; *** = 0.0001<; *P* < 0.001; ** = 0.001 < *P* <0.01. **(C)** RNA yield. Cultures of the *rne*^+^ and *rne*ΔMTS strains were grown to OD_600_ = 0.4 in LB medium. RNA was extracted from equal volumes of culture. Purified total RNA was eluted in equal volumes of water, and concentrations were determined by UV absorption at 260 nm. Average and standard deviation of RNA concentration from 3 independent experiments are shown. **(D)** Ribosomal RNA levels. Equal volumes of total RNA (Fig 1C) separated by agarose gel electrophoresis and staining with SybrGreen. Levels of 16S and 23S are comparable in the 2 strains, whereas there is 30% more Low Molecular Weigh RNA (LMW RNA) in the *rne*ΔMTS strain as estimated by quantification of fluorescence levels (Image Lab, Bio-Rad). **(E)** Polysome profiles. Clarified cell lysates prepared from equal volumes of cell cultures grown to OD_600_ = 0.4 in LB medium were fractionated by velocity sedimentation on 10%–40% sucrose gradients. Sedimentation is from left to right. Upper panel, *rne*^+^ strain; lower panel, *rne*ΔMTS strain. Peaks corresponding to 30S and 50S ribosomal subunits, 70S ribosomes and polysomes are indicated. 20S and 40S particles in the *rne*ΔMTS strain are indicated. The ratio of 70S Monosomes (M) to 70S Monosomes + Polysomes (P) was measured by integrating the area of the profile corresponding to monosomes or polysomes. The average and standard deviation of the M/M+P of 4 biological replicates is shown below each profile. The data underlying the graphs shown in Fig 1B, 1C and 1E can be found in S1 and S2 Data, respectively. Uncropped gel of Fig 1D can be found in S1 Raw Images.

We previously observed that the *rne*ΔMTS strain, which expresses ncRNase E, grows at approximately 80% of the rate of the isogenic *rne*^+^ strain expressing imRNase E [23]. To investigate the slow growth rate, we first analyzed cell shape and size. Visual inspection of the micrographs in Fig 1A shows no obvious morphological difference between the *rne*ΔMTS and *rne*^+^ strains. This result suggests that the slower growth rate is not due to defective cell wall synthesis or cell division since the morphology is normal. Next, we measured cell size. In LB medium, there is a small decrease in cell length and width in the mutant strain that results in about a 10% decrease in cell size (Fig 1B and S1 Data). Similar results were obtained in MOPS-glycerol medium, although the difference in cell width is negligible. From these results, we conclude that the slower rate of growth of the *rne*ΔMTS strain correlates with a small decrease in cell size, which is consistent with the known correlation between growth rate and cell size in *E*. *coli* [40].

During the preparation of RNA for transcriptome analyses [23], we noticed an approximately 60% increase in Low Molecular Weight (LMW) RNA in the *rne*ΔMTS strain during growth on minimal glucose medium (S1 Fig). When we extracted total RNA from exponentially growing strains in LB medium, we consistently obtained about 50% more RNA from the *rne*ΔMTS strain (Fig 1C and S2 Data). Since RNA was extracted from cultures grown to the same density (OD_600_ = 0.4) and there is only a small difference in cell size between the *rne*ΔMTS and *rne*^+^ strains, these results show a significant increase in total RNA levels in the *rne*ΔMTS mutant strain. We fractionated total RNA on an agarose gel by loading RNA extracted from equal volumes of cultures grown to the same density (Fig 1D). The levels of 23S and 16S rRNA are comparable, whereas the level of LMW RNA is about 30% higher in the mutant strain. The results in Fig 1C and 1D show that the 50% increase in total RNA is at least partly due to an increase in LMW RNA. Comparison of the data in Figs 1D and S1 shows that the percent increase in LMW RNA in the mutant strain depends on growth medium. Although we have previously reported a slowdown in mRNA degradation in the *rne*ΔMTS strain [23], it seems unlikely that the accumulation of mRNA degradation intermediates could by themselves explain the large increase in LMW RNA.

Comparable levels of 23S and 16S rRNA in the *rne*^+^ and *rne*ΔMTS strains strongly suggests that ribosome content in the mutant and wild-type strains are comparable. Nevertheless, the slow growth phenotype could be due to a defect in translation resulting in lower protein synthesis rates. We therefore analyzed polyribosome profiles by velocity sedimentation on sucrose gradients to compare the level of 70S ribosomes to polyribosomes. Fig 1E shows representative profiles from the *rne*^+^ and *rne*ΔMTS strains. Quantification of 4 biological replicates from each strain shows that there is no significant difference in the ratio of 70S monosomes to 70S monosomes plus polysomes (M/M+P), thus arguing against a defect in translation; data available in S2 Data. However, the appearance of aberrant particles in the mutant strain with sedimentation coefficients of approximately 20S and 40S is striking. This result suggests a defect in ribosome assembly in the *rne*ΔMTS strain that could explain the slow growth phenotype.

### Ribosomal RNA composition of 20S and 40S particles

**Fig 2:**
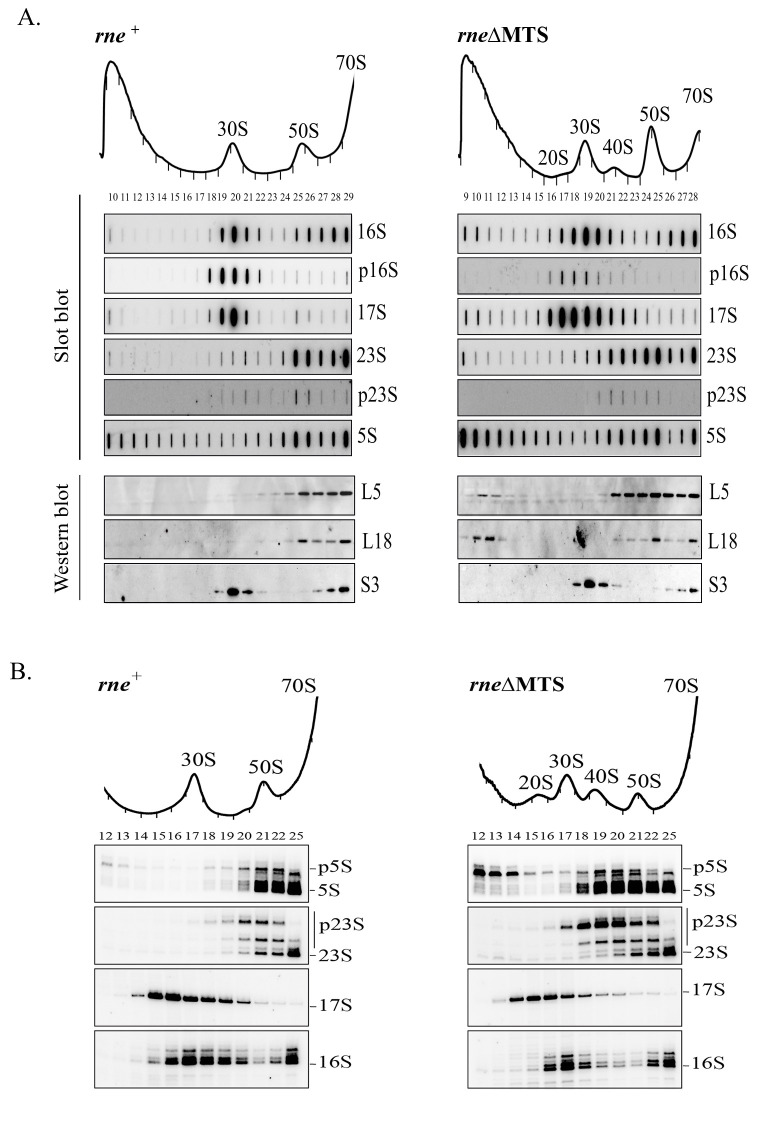
RNA content of ribosomal particles. Equal volumes of clarified cell lysates from *rne*^+^ (left) and *rne*ΔMTS (right) strains were fractionated by velocity sedimentation. Conditions were optimized for separation in the range of 20S to 70S. Sucrose gradient fraction numbers are indicated below the UV absorption profiles. **(A)** RNA from the sucrose gradient (fractions 10 to 29 for *rne*^+^ strain and fractions 9 to 28 for *rne*ΔMTS strain) was analyzed by slot blots using oligonucleotides specific to the RNA species indicated to the right of each panel. Protein from the sucrose gradient (fractions 10 to 28 for *rne*^+^ strain and fractions 9 to 27 for *rne*ΔMTS strain) was analyzed by western blotting using antibodies against the ribosomal proteins indicated to the right of each panel. **(B)** RNA from the sucrose gradient fractions was analyzed by primer extensions using [^32^P] end-labelled oligonucleotides specific to the 5′ ends of 5S, 23S, 17S, and 16S rRNA. After extension by reverse transcriptase, the products were separated by denaturing gel electrophoresis. The 5′ end of mature rRNA and that of the prominent precursors are indicated to the right of each panel. Bands located between the p5S and 5S ends correspond to 5S+1 and 5S+2 species. Uncropped slot blots, western blots, and gels of Fig 2A and 2B can be found in S1 Raw Images.

To characterize the RNA composition of the 20S and 40S particles in the *rne*ΔMTS strain, sucrose gradient sedimentation was optimized to resolve the 20S to 70S region. RNA extracted from each sucrose gradient fraction was analyzed by slot blots probed with oligonucleotides specific to 17S, p16S, 16S, p23S, 23S, and 5S rRNAs (Fig 2A). For comparison, we have included an analysis of sucrose gradient fractions from the wild-type strain. For both strains, the 30S subunit (fractions 18/19/20) contains 17S, p16S, and 16S rRNA, and the 50S subunit (fractions 24/25/26) contains p23S, 23S, and 5S rRNA. 17S, p23S, and 9S rRNA are released from the primary rRNA transcript by RNase III cleavage. The 17S and 9S intermediates are cleaved by RNase E to produces p16S and p5S rRNA, respectively, which together with the p23S intermediate are trimmed at their 5′ and 3′ ends by a battery of ribonucleases to produce mature 16S, 23S, and 5S rRNA [28,30]. The presence of the 17S intermediate in the 30S subunit and the p23S and p5S intermediates in the 50S subunit was confirmed by primer extension (Fig 2B). As a control, we analyzed the 5′ ends of polyribosomal rRNA by primer extension (S2 Fig). As expected, mature 16S, 23S, and 5S rRNA were detected in polyribosomes. Detection of rRNA intermediates in the 30S and 50S subunits shows that these are newly synthesized particles containing immature rRNA [26,34]. In the *rne*ΔMTS strain, the 20S particle contains the 17S and p16S precursors of 16S rRNA; the 40S particle contains the p23S and p5S precursors of 23S and 5S rRNA, respectively (Fig 2B). The mapping of rRNA precursors in Figs 2B and S2 was further confirmed by 5′ RACE analyses (S3 Fig). Together, these results show that the 20S and 40S particles are intermediates in ribosome assembly as evidenced by the presence of precursor rRNA species.

### Detection of oligoadenylated ribosomal RNA in the *rne*ΔMTS strain

The presence of p5S rRNA in the *rne*ΔMTS strain in the LMW fractions at the top of the gradient is striking (Fig 2B). In addition, p5S rRNA cosediments with L5 and L18 (Fig 2A, western blots), which are r-proteins known to bind to 5S rRNA [41]. The cosedimentation of p5S with L5/L18 in the LMW fractions is specific to the *rne*ΔMTS strain since they are almost undetectable in the *rne*^+^ strain. As a control, the sedimentation of S3, a 30S r-protein, shows no differences in the *rne*^+^ and *rne*ΔMTS strains, suggesting low levels of most r-proteins in the LMW fractions. Taken together, these results suggest that a proportion of p5S rRNA that is complexed with the L5/L18 r-proteins fails to incorporate into mature 50S ribosomal subunit in the *rne*ΔMTS strain. Since p5S rRNA is the product of RNase E cleavage, these results show that the defect in ribosome assembly is not due to a defect in RNase E processing of the 9S rRNA precursor.

**Fig 3:**
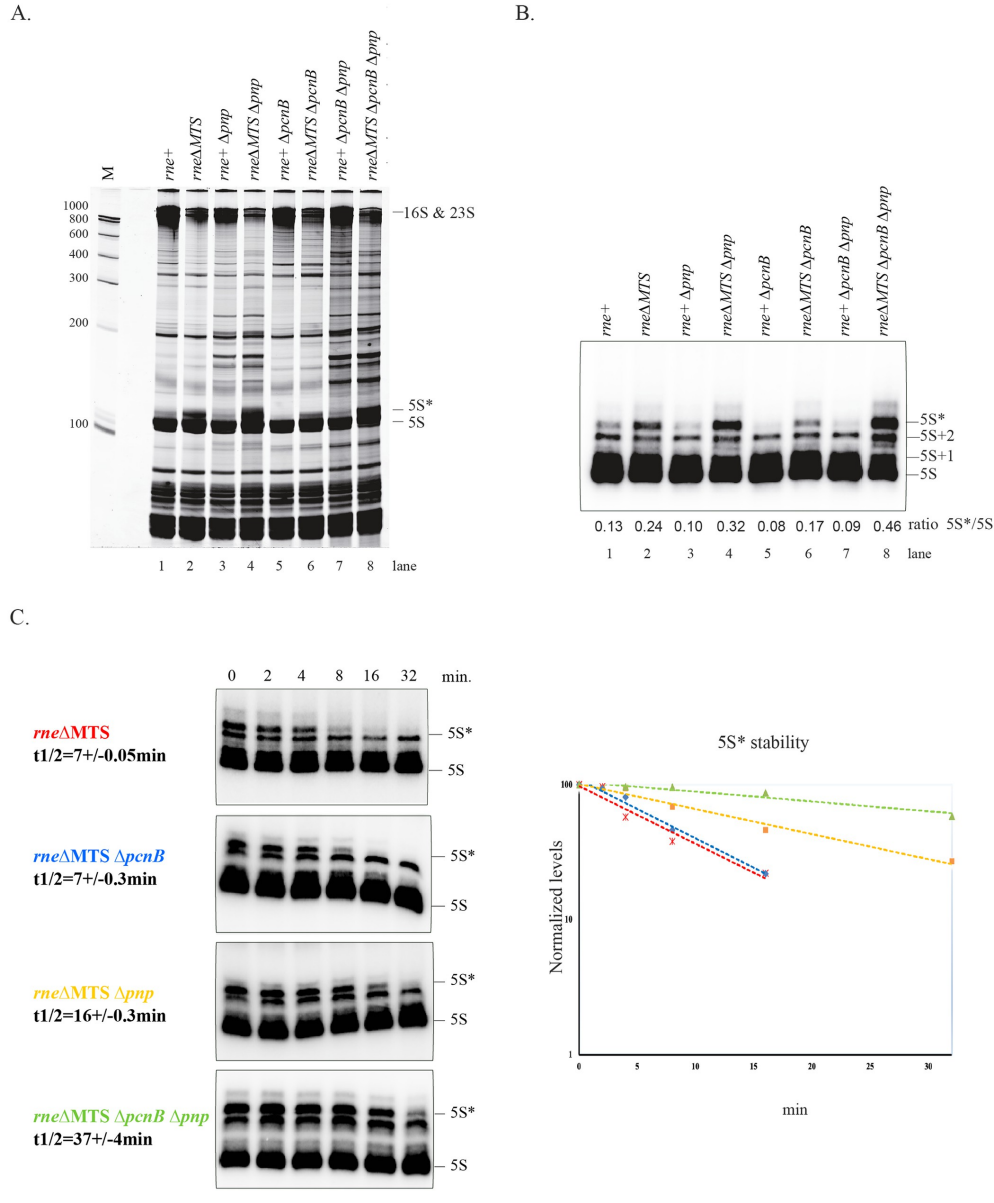
5S* rRNA is oligoadenylated form of p5S rRNA. **(A)** Total RNA (10 μg) was extracted from strains that were grown in LB at 37°C to OD_600_ = 0.4, separated on a denaturing polyacrylamide gel (10%, 7M urea) for 5 h at 300 V in 1× TBE and then stained with SybrGreen dye. **(B)** Total RNA (1 μg) was analyzed by primer extension with a probe specific for 5S rRNA. The position of mature 5S rRNA and its precursors are indicated on the right. The levels of 5S and 5S* were quantified by phosphorimaging. The 5S*/5S ratio is indicated at the bottom of each lane. **(C)** The decay of 5S* rRNA was measured after the inhibition of transcription by rifampicin (left panel). Strains and half-lives are indicated to the left of each panel. Semi-log plot of quantification by phosphoimaging used to calculate half-lives (right panel). Mean half-live and standard deviation were determined from 2 or 3 independent experiments for each strain. The data underlying the graph shown in Fig 3C can be found in S2 Data. Uncropped gels of Fig 3A-3C can be found in S1 Raw Images.

Separation of total RNA on denaturing polyacrylamide gels, which resolve small RNA species in the range of 50 to 500 nt, revealed a prominent RNA in the *rne*ΔMTS strain, migrating slightly slower than 5S rRNA, which we named 5S* rRNA. (Fig 3A, lane 2). Primer extension of total RNA with an oligonucleotide specific to 5S rRNA detected the presence of mature 5S rRNA 5′ ends as well as species with 5′ end extension (Fig 3B, lane 2). We have consistently seen 2 bands located between 5S and 5S* rRNA corresponding to species with 1 or 2 nt extensions, which agrees with work showing minor heterogeneity in the 5′ end of mature 5S rRNA [30,42]. We gel purified 5S rRNA from the *rne*^+^ and *rne*ΔMTS strains and 5S* rRNA from the *rne*ΔMTS strain and used RACE analyses to map the 5′ and 3′ ends of these molecules. A large proportion of the 5S rRNAs has a 5′ end corresponding to the mature molecule (S4A Fig). In contrast, most of the 5S* rRNAs has a 5′ AUU extension that corresponds to the p5S precursor, which is generated by RNase E cleavage of 9S rRNA precursor. Analysis of 3′ ends showed that nearly all 5S rRNA molecules have a mature 3′ end, whereas the 5S* molecules have heterogeneous 3′ ends (S4B Fig). A large proportion of these molecules have the 3′ CAA extension that corresponds to the p5S rRNA precursor as well as untemplated additions ranging from 1 to 4 nt. Untemplated 3′ additions are not detected in the *ΔpcnB* background, which lacks poly(A) polymerase activity (S4C Fig). The bands corresponding to 5S* rRNA (Fig 3A, lanes 2) therefore corresponds to a mixture of p5S rRNA and oligoadenylated p5S rRNA. 3′ end analysis of RNA extracted from sucrose gradient fractions showed that p5S as well as p23S rRNA are oligoadenylated in the 40S particles from the *rne*ΔMTS strain, whereas these RNAs were not oligoadenylated in 50S ribosomal subunits from the *rne*^+^ strain (S5 Fig).

Oligoadenylation of RNA 3′ ends by poly(A) polymerase, which is encoded by the *pcnB* gene, is known to facilitate exonucleolytic degradation by creating a single-stranded extension that acts as a binding site for exonucleases such as PNPase [43–45]. We therefore compared the electrophoretic profiles of total RNA in the mutant strains lacking PNPase and poly(A) polymerase (Fig 3A) and determined levels of 5S* rRNA by primer extension (Fig 3B). Isogeneic *rne*^*+*^ and *rne*ΔMTS strains were compared to reveal phenotypes specifically associated with the MTS deletion. In the *rne*^*+*^ strain, there are small amounts of 5S* rRNA. Levels expressed as the ratio of 5S*/5S shows that deletion of the genes encoding poly(A)polymerase and PNPase results in a large increase in 5S* rRNA. Deletion of the gene encoding PNPase alone also results in an increase in 5S* rRNA. Deletion of the gene encoding poly(A)polymerase results in the lower levels of 5S* rRNA, which is consistent with blockage of oligoadenylation. A kinetic analysis of 5S* rRNA decay after rifampicin treatment shows that p5S rRNA in the *rne*ΔMTS strain is degraded in a 3′ exonucleolytic pathway involving the activities of poly(A) polymerase and PNPase (Fig 3C and S2 Data). Since the effect of deleting both enzymes is cumulative, the poly(A)polymerase-dependent pathway likely involves RNase R activity (see [46–48]).

### 16S and 23S ribosomal RNA are fragmented by ncRNase E

**Fig 4:**
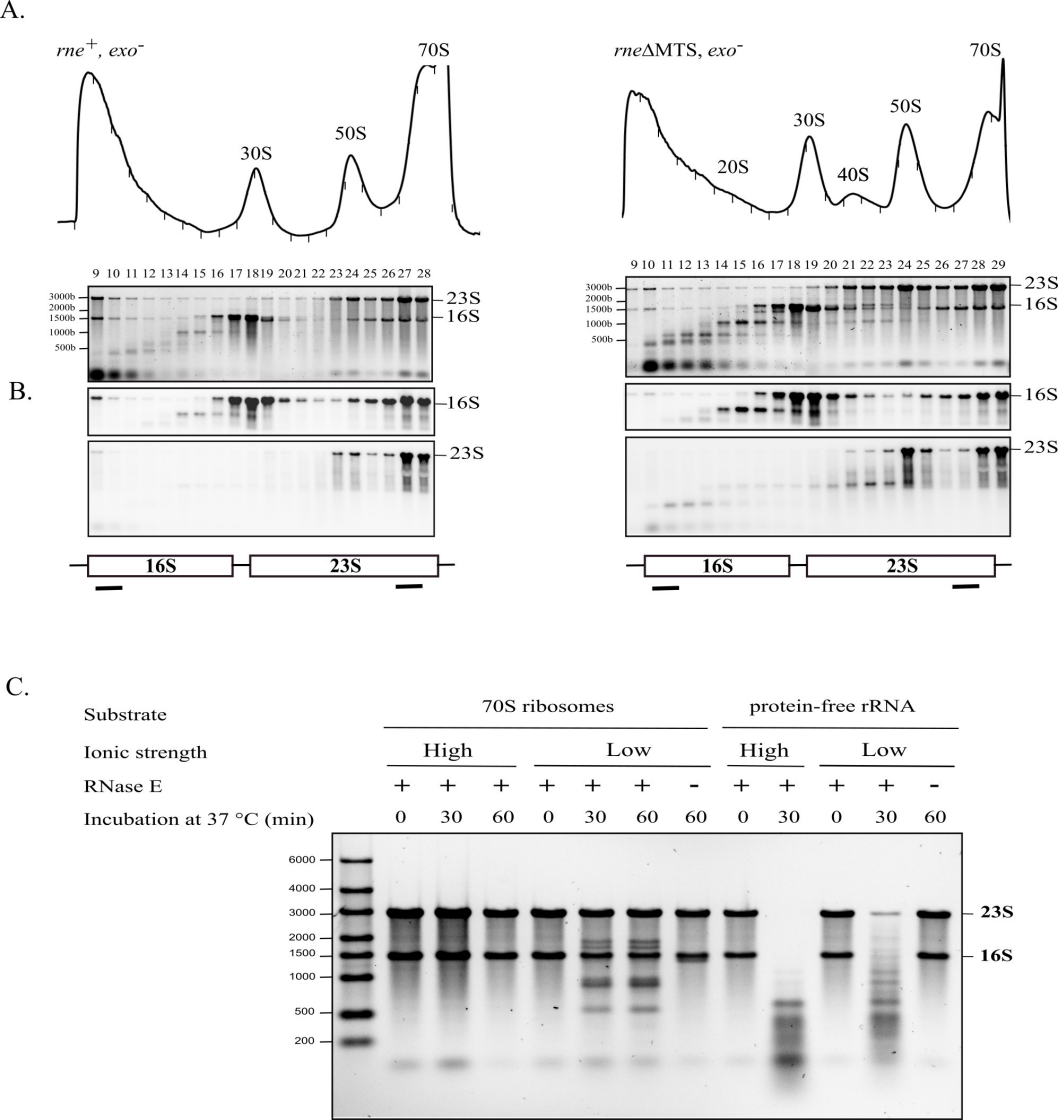
Identification of 16S and 23S rRNA fragments. **(A)** Equal volumes of cell lysates from the *rne*^+^, *exo*^*−*^ (left) and *rne*ΔMTS, *exo*^*−*^ (right) strains were separated by velocity sedimentation on 5% to 20% sucrose gradients. RNA from each fraction was separated by gel electrophoresis. **(B)** Northern blots with probes specific to the 5′ end of 16S rRNA and the 3′ end of 23S rRNA as indicated in the diagram at the bottom of each panel. **(C)** Degradation of rRNA in vitro. RNase E cleavage assays were performed with purified 70S ribosomes or protein-free rRNA. A representative experiment is presented. After incubation at the indicated temperature and times, RNA was extracted, separated on 1% agarose gels, and stained with SybrGreen. Each reaction contained 0.22 μM 70S ribosome or rRNA and 0.3 μM RNase E(1–598)-HIS6. Control lanes without RNase E (−) were also included. The position of the 23S and 16S rRNA are indicated to the right of the panel. Uncropped gels of Fig 4A and 4C and northern blots in Fig 4B can be found in S1 Raw Images.

Since previous work has shown that RNase E has an essential role in ribosome quality control [38], we asked if 16S and 23S rRNA are fragmented in the 20S and 40S particles, respectively. To identify internal RNase E cleavages in 16S and 23S rRNA, we used an *exo*^*−*^ strain background to knock down 3′ exonuclease activity and thereby increase the level of rRNA fragments. Although there are a large number of 3′ exonucleases in *E*. *coli*, RNase R and PNPase have a major role in the degradation of rRNA. Since inactivation of both genes encoding these enzymes is lethal, the *exo*^*−*^ background combines a knockout of the *rnr* gene with the *pnp-200* allele, which expresses a partially inactive variant of PNPase [49,50]. Fig 4A shows sedimentation profiles of ribosomes from strains in which the Δ*rnr* and *pnp-200* alleles were moved into the *rne*^+^ and *rne*ΔMTS strains. Total RNA was extracted from each fraction of the gradient and then separated by gel electrophoresis. As expected for both strains, full-length 23S and 16S rRNAs are found in 70S ribosomes and are present in 50S and 30S subunits, respectively. In the *rne*ΔMTS strain, the 20S particle, which is essentially devoid of intact 16S rRNA, contains shorter RNA species that are about 1000 and 500 nt in length. The 40S particle contains 23S rRNA as well as shorter RNA species that are about 1,700 and 1,000 nt in length. In addition, fragments of about 500 nt are conspicuous in the LMW RNA fractions of the *rne*ΔMTS strain.

Northern blots of RNA from the sucrose gradients were probed with oligonucleotides specific to the 5′ end of 16S rRNA and the 3′ end of 23S rRNA (Fig 4B). These blots show that the 1,000-nt RNA fragment in the 20S particles contains the 5′ end of 16S rRNA, and the 1,700-nt RNA fragment in the 40S particle contains the 3′ end of 23S rRNA. These results strongly suggest that rRNA in the 20S and 40S particles is fragmented by endonucleolytic cleavage in the body of 16S and 23S rRNA.

### In vitro cleavage of ribosomal RNA by RNase E

We tested the activity of RNase E on ribosomes or rRNA in vitro (Fig 4C). In a high ionic strength buffer, which is necessary for stability 70S ribosomes, rRNA is resistant to RNase E cleavage. Lanes at the right of the panel show that RNase E readily degrades protein-free rRNA in the high ionic strength buffer. Digestion, which results in a smear of fragments less than approximately 600 nt in length, shows that rRNA has a large number of RNase E cleavage sites. Resistance of ribosomes to cleavage in the high ionic strength buffer shows that rRNA secondary and tertiary interactions and r-proteins protect rRNA from RNase E cleavage. The cleavage of protein-free rRNA by RNase E is slower in the low ionic strength buffer due to the limiting amount of Mg^++^, which is necessary for RNase E activity. In the low ionic strength buffer, rRNA in ribosomes is nicked by RNase E to give a series of fragments ranging from 500 to 2,000 nt in length. From these results, we conclude that a subset of RNase E cleavage sites is accessible when the ribosome is destabilized in the low ionic strength buffer. Since low ionic strength dissociates the 30S and 50S subunits, RNase E cleavage could involve exposure of the subunit interfaces as has been described in previous work on ribosome degradation under conditions of nutrient starvation [36].

### Mapping RNase E cleavage sites in ribosomal RNA

**Fig 5:**
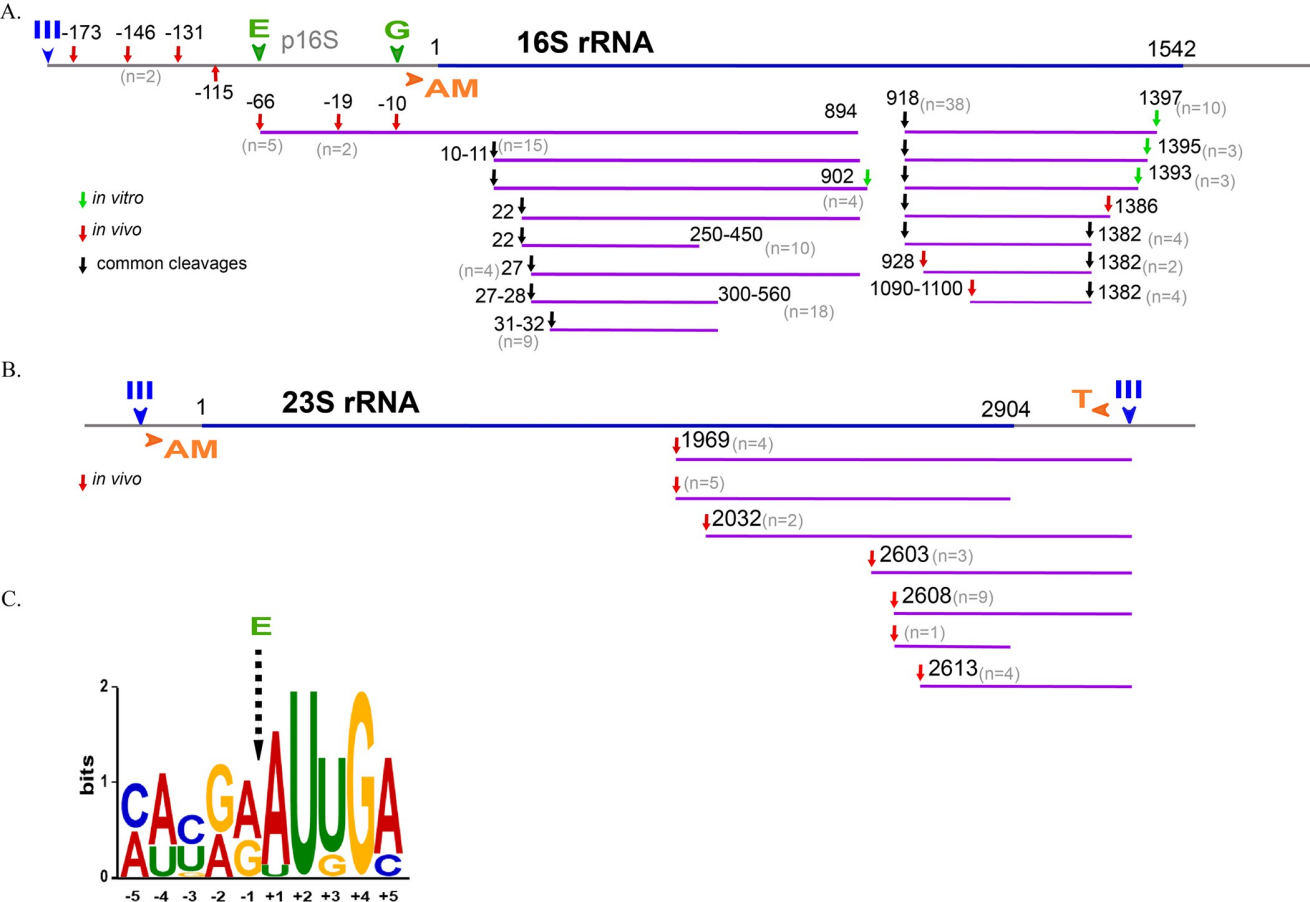
Mapping of RNase E cleavage sites. RNA fragments from the gels in Fig 4 were extracted, purified, and circularized, and the region containing the 3′-5′ junction was PCR amplified (cRACE), as indicated in S6 Fig. After cloning the PCR fragments into a plasmid vector, the 3′-5′ junction was sequenced and the ends aligned with the sequence of 16S or 23S rRNA. In the diagrams representing 16S and 23S rRNA, III (blue), E (green), G (green), AM (orange), and T (orange) represent, respectively, rRNA processing sites for RNase III, RNase E, and RNase G, which are endoribonucleases and RNase AM and RNase T, which are exoribonucleases that trim intermediates to the final mature species. **(A and B)** Identification of cleavage sites in 16S rRNA and 23S rRNA. The color-coded key indicates cleavages that were mapped in vivo, in vitro, or both in vivo and in vitro. The number of times a site was sequenced is indicated (n). The data underlying the schemes shown in Fig 5A and 5B can be found in S1 Table. **(C)** Consensus sequence of rRNA cleavage sites that were mapped in vivo.

Using cRACE (circular Rapid Amplification of cDNA Ends), we mapped 16S and 23S rRNA cleavage sites in vivo in the *rne*ΔMTS*-exo*^*−*^ strain and in vitro using purified RNase E and ribosomes. The strategy employed in this analysis is described in S6 Fig. S1 Table, which is a tabulation of the cRACE results, shows that the 3′ ends of in vivo fragments often contain noncoded oligo(A) additions. Fig 5A and 5B are schematic diagrams indicating RNase E cleavage sites mapped by cRACE. The frequency (n) represents the number of times an end was sequenced. The color-coded key indicates cleavages that were mapped in vivo, in vitro, or both in vivo and in vitro. Cleavages in vivo in the +22 to +32 region of 16S rRNA results in a nested set of fragments with raggedy 3′ ends that are likely due to partial degradation by residual 3′ exonuclease activity in the *rne*ΔMTS*-exo*^*−*^ strain. In Fig 5A, many of the internal cleavages in 16S rRNA were detected both in vivo and in vitro, which validates the in vitro cleavage of partially unfolded ribosomes by RNase E as a faithful representation of cleavage in vivo. Fig 5C shows the consensus sequence of rRNA cleavage sites that were mapped in vivo. The sequence is similar to the genome-wide consensus obtained from 22,000 RNase E sites in *Salmonella* mRNA, including the highly conserved U at position +2. [51]. These results are strong circumstantial evidence that ncRNase is responsible for cleavages of rRNA in the aberrant 20S and 40S particles.

### Proteomic analysis of the 20S and 40S ribosomal particles

**Fig 6:**
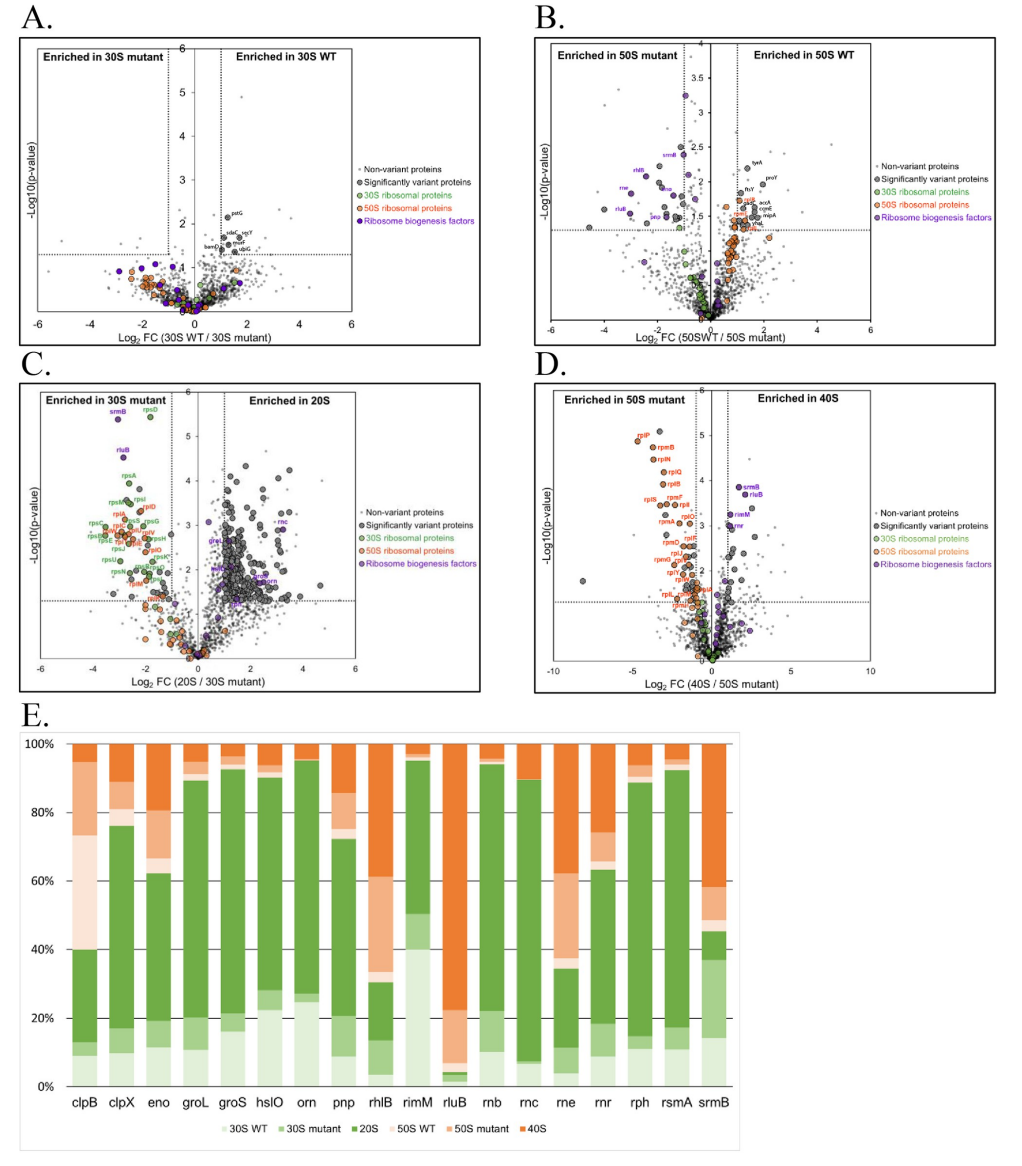
Protein composition of aberrant intermediates in ribosome assembly. Protein content of the ribosomal particles from *rne*^+^ and *rne*ΔMTS strains. Extracted proteins from sucrose gradient fractions were identified and quantified using a label-free quantitative mass spectrometry approach. Volcano plots showing significantly variant proteins (striped plots) in 30S particles from *rne*^+^ versus *rne*ΔMTS strains (**A**), in 50S particles from *rne*^+^ versus *rne*ΔMTS strains (**B**), in 20S versus 30S particles from *rne*ΔMTS strains (**C**), and in 40S versus 50S particles from *rne*ΔMTS strains (**D**) are presented. An unpaired bilateral Student *t* test with equal variance was used. Variant significance thresholds are represented by an absolute log2-transformed fold-change (FC) greater than 1 and a -log10-transformed (*p*-value) greater than 1.3 (see Materials and methods). Small subunit proteins (green), large subunit proteins (orange), and ribosome biogenesis factors (purple) are indicated. **(E**) Abundance levels of the quantified factors involved in ribosome biogenesis are represented as a percentage in 30S particle from *rne*^+^ strain (light green), in 30S particle from *rne*ΔMTS strain (medium green), in 20S particle (dark green), 50S particle from *rne*^+^ strain (light orange), in 50S particle from *rne*ΔMTS strain (medium orange), and in 40S particle (dark orange). The data underlying the graph shown in Fig 6 can be found in S2 Table.

We next analyzed protein content of the 20S and 40S particles from the *rne*ΔMTS strain. Proteins from sucrose gradient fractions corresponding to these particles as well as to the 50S and 30S subunits of the *rne*^+^ and *rne*ΔMTS strains were extracted, digested with trypsin, and then subjected to chromatography tandem mass spectrometry (nanoLC-MS/MS), leading to the identification and quantification of 1,286 proteins (S2 Table). To evaluate changes in protein compositions, pairwise comparisons based on MS intensity values were performed for each quantified protein, firstly, between *rne*^+^ and *rne*ΔMTS strains for 30S and 50S particles, secondly, between 20S and 30S particles as well as 40S and 50S particles in *rne*ΔMTS strain. Variant proteins were selected based on their significant protein abundance variations between the compared ribosomal particles (fold-change (FC) > 2 and < 0.5, and Student *t* test *P* < 0.05). Volcano plots in Fig 6A and 6B (S2 Table) show that composition of the 30S and 50S particles is globally the same in the 2 strains. The wild-type 30S and 50S particles are enriched in integral and associated membrane proteins (pstG, secY, sdaC, bamD, murF, ubiG, proY, ccmE, gadC, mipA, ftsY, and accY) [52,53]. Since the preparation of lysates for sucrose gradient analysis involves the use of sodium deoxycholate to solubilize membrane-associated ribosomes, an interaction of imRNase E as part of detergent micelles containing other membrane proteins with the ribosomal subunits could account for the detection of membrane proteins. In *the rne*ΔMTS strain, 17 small subunit proteins and 21 large subunit proteins are significantly underrepresented in the 20S and 40S particles, respectively (Fig 6C and 6D and S2 Table).

50S particles from the *rne*ΔMTS strain are enriched in ncRNase E, PNPase, RhlB, and enolase, which are components of the RNA degradosome, as well as SrmB and RluB (Fig 6B). SrmB is a DEAD-box RNA helicase that acts early in the assembly of the 50S subunit; RluB is a pseudouridine synthetase that acts late in the assembly of the 50S subunit [52,53]. Since it is likely that the gradient fractions analyzed here contain a mixture of particles (see Discussion), these results suggest that the 50S fraction from the *rne*ΔMTS contains a proportion of immature/defective particles whose degradation is initiated by the associated ncRNase E. 40S particles (Fig 6D) are also enriched in SrmB and RluB as well as RNase R and RimM, which is a factor involved in the assembly of the 30S subunit [52,53]. The enrichment of RimM suggests a noncanonical interaction with aberrant 40S particles. The 20S particle is enriched for RNase III, RNase PH, and oligoribonuclease (Fig 6C and 6E) as well as the protein chaperones GroEL-GroES, which have been shown to have a role in assembly of the 50S ribosomal subunit [54]. Oddly, in the *rne*ΔMTS strain, the 30S fraction is enriched in a subset of large subunit proteins (Fig 6C). Since the particle peaks in velocity sedimentation on sucrose gradients are broad due to diffusion, the trailing edge of faster sedimenting particles overlaps the leading edge of slower sedimenting particles, and the 40S particle is a heterogeneous mixture of degradation intermediates (see Discussion), the detection of large subunit proteins in the 30S subunit is likely due to contamination by 40S degradation intermediates. Proteins involved in ribosome assembly including enzymes that modify rRNA, ribonucleases, DEAD-box RNA helicases, the GroEL protein chaperone, and the ClpXP protease are associated with the 20S and 40S particles, and most of these factors are underrepresented in the 30S and 50S ribosomal subunits (Fig 6E and S2 Table). The underrepresented r-proteins and the associated ribosome assembly factors are supporting evidence for the conclusion that the 20S and 40S particles are aberrant dead-end intermediates in ribosome assembly.

## Discussion

Here we have shown that the *E*. *coli rneΔMTS* strain expressing ncRNase E has an abnormal ribosome profile with high levels of 20S and 40S particles. 5′ and 3′ end analysis showed that the particles contain precursors of 16S, 23S, and 5S rRNA, thus supporting the conclusion that they are intermediates in ribosome assembly as opposed to intermediates in the degradation of mature ribosomal particles. rRNA in the 20S and 40S particles is fragmented by ncRNase E cleavage within the 16S and 23S sequences. Mapping of ncRNase E cleavages in the 20S and 40S particles revealed sites whose sequences correspond to the consensus previously determined by genome-wide mapping of cleavages in *Salmonella* [55]. In vitro experiments with purified RNase E and ribosomes showed that properly folded ribosomes are resistant to RNase E cleavage, whereas protein-free rRNA is readily degraded by RNase E. rRNA in ribosomes that are partially unfolded in vitro under low ionic strength conditions is cleaved by RNase E at sites that were mapped in vivo. From these results, we conclude that rRNA cleavage sites in intact 70S ribosomes are sequestered by rRNA folding and r-protein binding. Furthermore, as has been described in work on ribosome degradation under conditions of nutrient limitation, the 30S to 50S interface of the 70S ribosome could sequester rRNA from RNase E cleavage that has been proposed to initiate ribosome degradation [36–38].

In the *rneΔMTS* strain, fragments of 16S and 23 S rRNA as well as p5S rRNA have 3′ untemplated oligo(A) extensions. Oligoadenylated p5S rRNA migrates electrophoretically as a distinct species that we named 5S*. In vivo results with mutant strains showed that 3′ exonucleolytic degradation of 5S* rRNA involves the activities of PNPase and poly(A) polymerase. Measurements of 5S* degradation after rifampicin treatment showed an approximately 5-fold increase in half-life in a *pnp*^*−*^-*pcnB*^*−*^ strain relative to the isogenic *rneΔMTS* control. It is also noteworthy that the exonucleolytic degradation pathway for 5S* rRNA is the same as previously described for several sRNA molecules as well as mRNA degradation intermediates containing REP elements [46,47,56,57]. Our velocity sedimentation results show that, in the LMW fraction at the top of the sucrose gradient, there are significant amounts of 5S rRNA precursors that cosediment with r-proteins L5 and L18, which are known to bind to 5S rRNA [41]. These results suggest that the p5S-L5-L18 complex accumulates as an intermediate in the *rneΔMTS* strain and that its failure to incorporate into the 50S ribosomal subunit triggers degradation.

The 20S and 40S particles are, respectively, nominally equivalent to the p_1_30S intermediate, which sediments as a 21S particle, and the p_2_50S, which sediments as a 43S particle. Nevertheless, our proteomics analysis showing that 17 small subunit proteins and 21 large subunit proteins are underrepresented in the 20S and 40S particles, respectively, is inconsistent with their identification as bona fide intermediates in ribosome assembly. We believe they are defective intermediates that accumulate due to damage by RNase E cleavage, which blocks correct rRNA folding and r-protein binding. Furthermore, it is unlikely that the particles in the 20S and 40S sucrose gradient fractions are homogeneous in composition. Recent analysis of sucrose gradient fractions in the trailing edge of the 30S peak in a wild-type strain showed that they contain a heterogeneous mixture of intermediates in ribosome assembly [58]. Similar results with intermediates in assembly of the 50S particle has led to the conclusion that ribosome assembly involves cooperative rRNA folding blocks that correspond to structural domains in the mature 30S and 50S ribosomal subunits and that there are multiple parallel pathways leading to mature 30S and 50S ribosomal subunits [27,35].

Considering the large number of ncRNase E cleavages of rRNA that we have mapped in the *rneΔMTS* strain, we suspect that there are multiple pathways for the interference of ncRNase E with ribosome assembly. Although RNase E cleavage sites are single-stranded, the enzyme has the capacity to bind to structured RNA [59,60]. We therefore propose that ncRNase E competes directly with cotranscriptional r-protein binding resulting in misfolded intermediates lacking r-proteins. This proposal is consistent with the finding that ncRNase E is distributed uniformly through the interior of the cell including the region in the center of the cell where the nucleoid is located [13]. These defective intermediates are then cleaved by ncRNase E, which initiates their degradation. Although cotranscriptional interference with r-protein binding might be expected to trigger rho-dependent transcription termination, the rRNA transcription elongation complex is insensitive to rho-mediated termination [32,61]. We believe that ncRNase E interference and rRNA cleavage are stochastic processes leading to a large number of different dead-end intermediates. The association of ribosome assembly factors with the 20S and 40S particles suggest that these factors are trying to “rescue” defective intermediates. However, the degradation of rRNA in these particles suggests that the damage is mostly irreversible.

**Fig 7:**
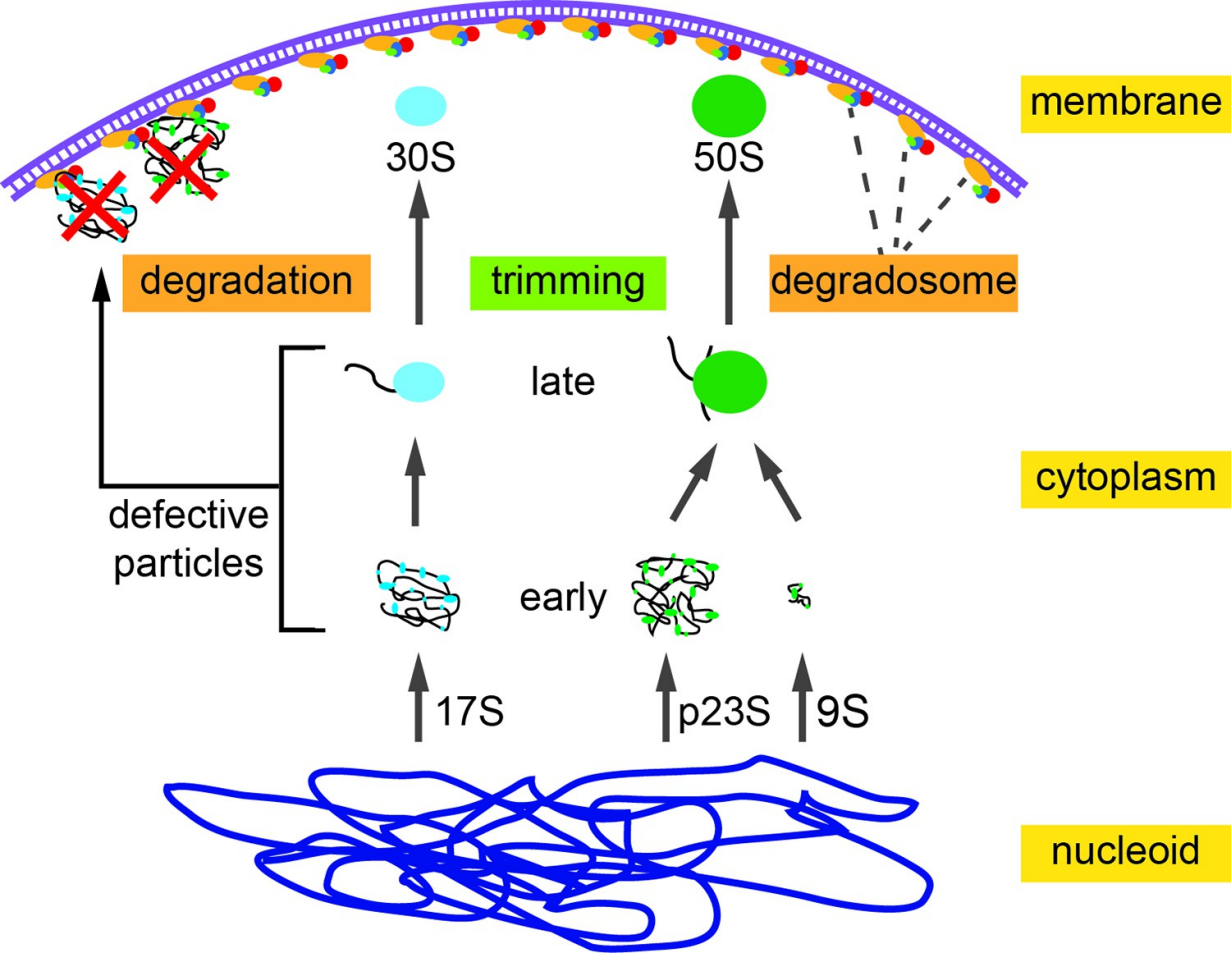
Quality control of ribosome assembly by the membrane-attached RNA degradosome. Cartoon depicting the synthesis of rRNA in the nucleoid, the release of early intermediates in ribosome assembly from nucleoid, maturation of late intermediates in the cytoplasm, and trimming of 17S and 9S rRNA by the membrane-attached RNA degradosome. Defective ribosomal particles are degraded by the membrane attached RNA degradosome. In this model, compartmentalization of ribosome assembly to the interior of the cell and the RNA degradosome on the inner cytoplasmic membrane shields intermediates in ribosome assembly from degradation, thus avoiding wasteful turnover of rRNA. Defective particles can be either newly synthesized intermediates that have failed to properly fold or mature ribosomal subunits that are inactive (see Discussion).

Our results strongly suggest that quality control of ribosomes is mediated by imRNase E. Fig 7 is a cartoon depicting how the compartmentalization of the RNA degradosome to the inner cytoplasmic membrane protects partially unfolded intermediates in ribosome assembly from wasteful degradation. In this model, membrane attached RNA degradosomes are involved in the “trimming” of 17S rRNA to p16S rRNA and 9S rRNA to p5S rRNA [62–64]. We propose that trimming of intermediates in ribosome assembly on the inner cytoplasmic membrane occurs after the subunits are properly folded and contain a full complement of r-proteins. This leads to the suggestion that the membrane attached RNA degradosome acts as a sensor that discriminates between properly folded, functional ribosomes and partially unfolded, inactive ribosomes that are degraded by the membrane-attached RNA degradosome. However, we believe that the interference of ncRNase E with ribosome assembly is likely to be mostly cotranscriptional in the nucleoid and that normal ribosome quality control starts after intermediates are released from the nucleoid.

Ribosome biogenesis is a major activity in growing cells. The time it takes for a cell to double is directly related to the time it takes to double ribosome content. Since rRNA synthesis is the limiting step in ribosome biogenesis [65], the wasteful degradation of rRNA likely explains the slower rate of growth of the *rneΔMTS* strain compared to the *rne*^*+*^ strain [6,23]. Enzymes involved in rRNA and mRNA degradation are the same [36–39,66]. Since recent work has shown the importance of competition between RNase E substrates in setting rates of mRNA degradation [67,68], competition between rRNA and mRNA degradation could explain the global slowdown in mRNA degradation in the *rne(ΔMTS)* strain [23]. The work reported here shows that membrane attachment of RNase E as a component of the RNA degradosome is necessary to avoid a futile cycle of wasteful degradation of intermediates in ribosome assembly. Conservation of membrane-associated RNase E throughout the β- and γ-Proteobacteria is likely due to selective pressure to avoid interference with ribosome assembly.

RNase E homologues in the α-Proteobacteria lack identifiable MTS sequences [2,17]. Recent work, principally in *Caulobacter crescentus*, has shown that these proteins are not attached to the inner cytoplasmic membrane. The RNA degradosome of *C*. *crescentus* is localized to the interior of the cell in condensates known as BR-bodies (Bacterial Ribonucleoprotein-bodies) that have properties similar to eukaryotic stress granules and P-bodies [69,70]. Assembly of BR-bodies is dynamic and requires RNA substrate as evidenced by rifampicin treatment. The endoribonuclease activity of RNase E is necessary for the disassembly of BR-bodies as evidenced by catalytically inactive variants of the enzyme. The intrinsically unstructured C-terminal region of RNase E, which is conserved in the α-Proteobacteria, is necessary and sufficient for BR-body formation. Characterization of the RNA content of *Caulobacter* BR-bodies showed that they are enriched in mRNAs and that rRNA and tRNA are excluded [71]. It was thus proposed that the *Caulobacter* BR-body is a compartment nucleated by the RNA degradosome in which mRNA is degraded. Selective permeability of the BR-body results in the enrichment of mRNA and mRNA decay intermediates, thus increasing their concentration and driving degradation to nucleotides, which is important for maintaining nucleotide pools for transcription and DNA replication in growing cells. Importantly, BR-bodies form a compartment that is distinct from the nucleoid and cytoplasm. These results suggest that intermediates in ribosome assembly in *Caulobacter* are protected from cleavage by *Caulobacter* RNase E due to the sequestration of the RNA degradosome into condensates that exclude ribosome precursors, ribosomes, and polysomes.

Transcription and mRNA degradation in *E*. *coli* and *C*. *crescentus* are physically separated in membraneless compartments. These bacteria, which are separated by billions of years of evolution, use different strategies to achieve similar outcomes [17]. Short-lived RNA degradosome puncta on the inner cytoplasmic membrane of *E*. *coli* are centers of mRNA degradation. The membrane attached RNA degradosome is also involved in the processing of rRNA and quality control of ribosomes. The physical separation of the RNA degradosome on the inner membrane from early steps in ribosome assembly in the nucleoid is necessary to prevent degradation of intermediates in ribosome assembly. The compartmentalization of RNA degradosomes in *Caulobacter* BR-bodies has functions similar to the membrane attachment of *E*. *coli* RNase E. BR-bodies are condensates in which the RNA degradosome and ribosome-free mRNA are concentrated, thus driving degradation to nucleotides. BR-bodies exclude rRNA, ribosomes, and polysomes, thus segregating ribosome assembly from mRNA degradation. Compartmentalization of the mRNA degrading machinery in *E*. *coli* and *Caulobacter* is a fascinating example of evolution in which different cellular organizations result in solutions to similar problems involving the accessibility of RNA substrates to the RNA degradosome and the concerted degradation of mRNA to nucleotides.

## Materials and methods

### Bacterial strains and growth

Bacterial strains, plasmids, and primers used in this study are listed in Tables 1 and 2. Bacteria were grown either in LB or MOPS media prepared as described [72,73] at 180 rotations per minute (rpm) with normal aeration or agar plates at 37°C. All mutant strains were constructed using the lambda-Red system as described in [74]. After allele substitution into the chromosome using an antibiotic resistance cassette, the constructs were genetically purified by bacteriophage phage P1 transduction, and the cassettes were removed using FLP recombinase resulting in an *frt* (FLP recognition target) scar. All constructs were validated by sequencing PCR products amplified from chromosomal DNA.

**Table 1:** Strains and plasmids.

**Strain**	**Genotype**	**Reference**
NCM3416	*E*. *coli* K12, F-, λ-, *rph+*, *zib-207*::Tn*10*	[80]
MG1655	F^−^, λ^−^, *rph*^*−*^	[81]
MBS106	NCM3416, *rne-frt*	[23]
MBS157	NCM3416, *rne*Δ*MTS-frt*	[23]
BL21(DE3)	F^−^, *omp*T, *hsd*S_B_ (r_B_^−^, m_B_^−^), *dcm*, *gal*, λ(DE3)	[82]
Kti658	MG1655, *rne*Δ*MTS-frt*	[23]
Kti665	NCM3416 *rne*+ Δ*pcnB*	This work.
Kti669	NCM3416 *rneΔMTS* Δ*pcnB*	This work.
LHS-457	NCM3416 *rne*+ Δ*pcnB* Δ*pnp*	This work.
LHS-459	NCM3416 *rneΔMTS* Δ*pcnB* Δ*pnp*	This work.
SQM-5	NCM3416 *rne*+ Δ*pnp*	This work.
SQM-6	NCM3416 *rneΔMTS* Δ*pnp*	This work.
SLP-35	NCM3416 *rne*+ Δ*rnr*	This work.
SLP-36	NCM3416 [32]*rneΔMTS* Δ*rnr*	This work.
CA244	*rnr*^*−*^ *pnp-200 Cm*^*r*^	[50]
SLP-79	NCM3416 *rne*+ Δ*rnr pnp200-kn*	This work.
SLP-80	NCM3416 *rneΔMTS* Δ*rnr pnp200-kn*	This work.
Kti162	NCM3416, *rne-mcherry*	[12]
Kti513	NCM3416, *rneΔMTS-mcherry*	[12]
**Plasmid**	**Features**	**Reference**
pLP56-2	pET-*rne*(1–598)-his6	[23]

**Table 2:** Primers.

Name	Sequence (5′ to 3′)	Use
**LHO-183**	CGTATCTTCGAGTGCCCACA	Probe for 17S in slot blotting analysis [75]
**LHO-184**	GTGTTCACTCTTGAGACTTGG	Probe for p16S in slot blotting analysis
**LHO-187**	CGCTTAACCTCACAAC	Probe for p23S in slot blotting analysis [75]
**MBO-059**	ACTACCATCGGCGCTACGGC	Probe for 5S in slot blotting analysis
**OLP118**	TCCTCCCCGCTGAAAGTACT	Probe for 16S (431–450) in northern and slot blotting analysis
**OLP125**	GACTGGCGTCCACACTTCAA	Probe for 23S (2,131–2,150) in northern and slot blotting analysis
**RNA A3**	AUAUGCGCGAAUUCCUGUAGAACGAACACUAGAAGAAAG	RNA adapter for 5′ RACE analysis
**DNA B6**	GCGCGAATTCCTGTAGA	Primer sense to RNA A3 adapter for 5′ RACE analysis
**DNA E4**	GGCCGCTAAGAACAGTGAA	Primer antisense to E1 RNA adapter for 3′ RACE analysis [78]
**RNA E1**	(5′-P) UUCACUGUUCUUAGC GGCCGCAUGCUC (idT-3′)	RNA adapter for 3′ RACE analysis
**MBO-163**	CGGCGTTTCACTTCTGAGTTCGGC	5S rRNA primer extension and 5′ RACE
**LHO-117**	CCTTCATCGCCTCTGACTGCCA	23S rRNA primer extension, 5′ RACE and cRACE analyses
**LHO-124**	GCATGTGTTAGGCCTGCCGC	16S rRNA primer extension, 5′ RACE and cRACE analyses
**LHO-132**	GAACTCAGAAGTGAAACGCCG	Sense PCR primer for 5S 3′ RACE analysis
**LHO-150**	CGGGTGTGTAAGCGCAGCG	Sense PCR primer for 23S 3′ RACE analysis
**LHO-203**	GCAAGTCGAACGGTAACAGG	PCR primer for 16S 5′ end cRACE analysis
**LH0–204**	CTGGTCGTAAGGGCCATGATG	16S rRNA primer extension, cRACE analysis
**LH0–205**	CAGGGCTACACACGTGCTAC	PCR primer for 16S 3′ end cRACE analysis
**LHO-206**	GACGTGCTAATCTGCGATAAG	PCR primer for 23S 5′ end cRACE analysis
**LHO-207**	CTTCAACGTTCCTTCAGGACC	23S rRNA primer extension, cRACE analysis
**LHO-208**	GACGACGACGTTGATAGGCC	PCR primer for 23S 3′ end cRACE analysis

### Cell dimension measurements

Samples for microscopy were prepared as in [39]. Briefly, bacterial strains were grown to OD_600_ = 0.5 at 37°C with shaking in LB or MOPS medium supplemented with 0.5% glycerol and amino acids. Microscopy images were acquired on a Nikon Eclipse TI-E/B wide field epifluorescence microscope using phase contrast objective and were analyzed using Image J V.1.38 software. Statistical analysis and graphs were generated using GraphPad Prism, version 7.

### Polysome fractionation analysis

Polysomes fractionation analyses were performed as described [75–77] with some modifications. Briefly, overnight cultures diluted in fresh LB medium were cultured at 37°C to an OD_600nm_ of 0.4. To stop bacterial growth and avoid ribosomes/polysomes dissociation, 40 OD_600_ equivalent units were harvested by fast-chilling by placing the cultures directly in a cold flask on an ice-water bath with shaking for 3 min. After centrifugation at 6,000*g* for 15 min at 4°C (JA14 rotor-Beckman), the cell pellet is resuspended with cold lysis buffer (1 mg/ml lysozyme, 10 mM MgCl_2_, 60 mM KCl, 10 mM Tris–HCl (pH 8)). For complete lysis, cells were subjected to 2 freeze–thaw cycles. After the second freeze–thaw cycle, 0.3% of sodium deoxycholate anionic detergent (D6750_SIGMA) was added to solubilize the membrane proteins, and the lysate was clarified by centrifugation at 10,000*g* for 10 min at 4°C. To analyze polysome profiles, a constant volume of extract was layered onto an ultracentrifuge tube (tube 13.2 mL-Beckman Coulter SW-41) containing a continuous 10% to 40% (w/v) sucrose gradient prepared in the following buffer: (10 mM MgCl_2_, 20 mM Tris–HCl (pH 7.5), 100 mM NH_4_Cl, 2 mM dithiothreitol (DTT)) and centrifuged at 35,000 rpm for 3 h 30 at 4°C in an Optima XPN-80-Beckman Coulter ultracentrifuge. Sucrose gradients were analyzed on a density gradient fractionation system (ISCO UA-6 detector/Brandel Foxy Gradient) with continuous monitoring at 254 nm, allowing the various ribosomal peaks to be resolved. To compare levels of 70S monosomes (M) and polysomes (P) in different strains, areas under the curves corresponding M and P were measured using ImageJ software, and an M/M+P ratio was then calculated. To specifically analyze and resolve the ribosomal subunits, the extracts were layered onto a continuous 5% to 20% (w/v) sucrose gradient in the same buffer described above and centrifuged at 28,600 rpm for 7 h at 4°C in a Beckman SW-41 rotor. The collected fractions were subjected to RNA and/or protein analyses.

### Total RNA extraction

Two to 4 OD_600_ of bacterial cell cultures were mixed with 0.2 volume of stop solution (ethanol: phenol 95:5 v/v) and snap frozen in liquid nitrogen. Samples were thawed on ice and spun at 4,000 rpm for 15 min at 4°C, and the cell pellet was dissolved in 1 mL TRIzol (Invitrogen, #15596026). An equal volume of ethanol was added to the mixture, and total RNA was prepared using a Direct-zol, RNA MiniPrep Plus kit (Zymo Research) following the manufacturer’s instructions and DNase I digested using the DNase I provided in the same kit. RNA was eluted in 80 μl milliQ water (RNase-free), and RNA amount and purity were determined using a NanoDrop spectrophotometer.

### RNA isolation after fractionation on sucrose gradients

RNAs were extracted from sucrose gradient fractions by adding 1 volume of TRIzol and by using the Direct-zol RNA Miniprep Plus kits (ZYMO RESEARCH, #R2072). The RNAs were eluted in 60 μl of milliQ water (RNase-free) and subjected to DNase I digestion. After purification, the same amounts of RNA, unless indicated elsewhere, were used to perform primer extension analyses on the rRNAs as described below. Moreover, the same volume of RNA from sucrose fractions was also separated on native agarose gels (1%) (that do not contain formaldehyde), in 1X TBE buffer (10X TBE: 890 mM Tris base, 890 mM boric acid, 20 mM EDTA) for 3 h 30 at 50 V. After electrophoresis, the gels were either subjected to northern blotting as described below or stained with SYBR Safe stain (Invitrogen).

### Primer extension analysis

Primers specific to 5S, 16S, or 23S rRNA (2 pmol 5’ end-labelled) and 0.25 to 1 μg of RNA (total RNA or RNA extracted from sucrose gradient fractions) were denatured together in water for 5 min at 65°C and immediately quenched on ice for 5 min. DeoxyNTPs (50 μM), 1x first strand buffer, 5 mM DTT, 1 U/μl RNase inhibitor (Thermo Scientific), and 1 μl Superscript III reverse transcriptase (200 U, Invitrogen) were added to the denatured RNA and primer (20 μl reaction). Primer extension was allowed for 50 min at 55°C. After heat inactivation of the reverse transcriptase for 5 min at 85°C, samples were treated with 2 U RNase H (Thermo Scientific) at 37°C for 20 min. The resulting reaction (2 to 5 μl) was mixed with an RNA loading dye and resolved on a 6% PAA, 7 M urea sequencing gel along with the sequencing ladder. The sequencing ladder was obtained on a plasmid containing the 9S coding gene (*rrfB*) using USB Sequenase version 2.0 DNA polymerase (Affymetrix) following the supplier’s instructions. cDNA signals were visualized on a phosphoimager (Typhoon Trio- Amersham-Bioscience), and band intensities were quantified using MultiGauge software (Fujifilm).

### Northern blotting

DNase I-digested total RNA (5 to 10 μg) was denatured for 5 min at 95°C in RNA loading dye (95% formamide, 0.1% xylene cyanol, 0.1% bromophenol blue, 10 mM EDTA), chilled on ice for 2 min, then separated either on 6% denaturing PAA gels (7 M urea) or on 1% agarose gels (native conditions). The RNA was transferred to Hybond-XL membrane (GE Healthcare) by electroblotting at 50 V, for 1 h using 1X TBE buffer (10X TBE: 890 mM Tris base, 890 mM boric acid, 20 mM EDTA), then cross-linked to the membrane by UV crosslinking (120 kJ). The membranes were preincubated for 1 h with 15 ml of Roti-Hybri-Quick buffer (Roth) at 42°C, and then the radiolabeled probes were added and incubated ON. The membranes were rinsed with 5X SSC (20X SSC: 3 M sodium chloride, 0.3 M sodium citrate, SSC buffer contains in addition 0.1% SDS) to remove the nonhybridized probe, then washed 3 times at 42°C with SSC buffer (15 min with 5X SSC, 15 min with 1X SSC, and 15 min with 0.1X SSC). RNA signals were visualized on a phosphoimager (Typhoon Trio- Amersham-Bioscience), and band intensities were quantified using MultiGauge software (Fujifilm).

### Slot blot

The slot blots were generated as described in [23]. Cell extract from fractions collected after sucrose gradient fractionation (20 μl) was denatured in the presence of denaturing buffer (2.2 M formaldehyde, 50% formamide, 0.5 mM EDTA, 10 mM MOPS, 4 mM NaCl) and incubated at 65°C for 5 min. Samples were directly placed in a slot on a nylon membrane by vacuum filtration (Amersham Hybond-XL-GE Healthcare) using a transfer collector (PR648-Hoefer Slot Blot). The RNA present in the deposited extracts was irreversibly fixed to the membrane by ultraviolet treatment (120 kJ/cm^2^). The membranes were subsequently hybridized with radiolabeled oligonucleotide probes specific for the rRNAs as described above for northern blot.

### 5′ RACE

The 5′ ends of rRNAs were mapped using 5′ RACE (rapid amplification of cDNA ends) analysis following the protocol described [78]. First, total RNA or purified rRNAs with 5′ monophosphate ends were ligated to the 3′ hydroxyl group of an RNA oligonucleotide adapter, followed by reverse transcription with a gene-specific primer (RT primer) and subsequent PCR amplification using a 5′-adapter-specific primer and a gene-specific primer. Briefly, the RNA-adapter ligation was performed overnight at 17°C in the presence of 0.5 to 1 μg total RNA or purified 5S and 5S* rRNA, 21 pmol of RNA adapter (RNA A3), 10 units of T4 RNA ligase (Thermo Scientific), 1X RNA ligase buffer containing ATP, 15% DMSO, and 20 units of RNase Inhibitor in a 20-μl final reaction. After addition of 2 pmol of an RT primer, the reaction was adjusted to a final volume of 150 μl by adding milliQ water (RNase-free). Subsequently, the adapter-ligated RNA was extracted with 1 volume of phenol-chloroform-isoamyl alcohol (P:C:I) in PLG tubes. The aqueous phase was mixed with 3 volumes of a mixture of ethanol and sodium acetate at pH 5, ratio 29:1, to precipitate the RNAs. The adapter-ligated RNA were dissolved in 30 μl of milliQ water (RNase-free). Adapter-ligated RNA was converted to cDNA using an RT primer specific for each gene encoding the rRNAs and the Superscript III reverse transcriptase as described above (primer extension). After treatment with 1 unit of RNase H (Thermo Scientific), 2 μl of the cDNA samples were used as template for a PCR reaction using 1 unit of PHUSION DNA polymerase (Finnzymes), 1X GC buffer, 0.2 mM dNTPs, 3% DMSO, and 1 μM of the pair of oligonucleotides: the sense primer DNA b6, which anneals to the RNA-adapter sequence, and an antisense primer that anneals within the gene of interest (5S, 16S, or 23S). Following visualization on 3% agarose gels, PCR products were excised, purified, and then sequenced after cloning using the Zero Blunt TOPO PCR cloning kit (Invitrogen).

### 3′ RACE

Total RNA or purified RNA (0.5 to 1 μg) was first dephosphorylated using 1 U of thermosensitive Alkaline phosphatase FastAP (Thermo Scientific) in the presence of 10X AP buffer and 20 U of RNase inhibitor (Thermo Scientific) in a final volume reaction of 20 μl for 15 min at 37°C. Dephosphorylated RNA was subjected to P:C:I extraction and precipitation with 3 volumes of 30:1 ethanol/sodium acetate solution. The dephosphorylated RNA was ligated to an RNA adapter (RNA E1) ON at 17°C, P:C:I extracted and precipitated as described above. Ligated RNA (0.25 to 0.50 μg) was reverse transcribed in the presence of 5 pmol of E4 DNA primer (complementary to the E1 RNA adapter) using 200 U of Superscript III (Invitrogen) as described above. After treatment with 1 U of RNase H (Thermo Scientific), 2 μl of the cDNA samples were used as template for a PCR reaction using 1 U of PHUSION DNA polymerase (Finnzymes), 1X GC buffer, 0.2 mM dNTPs, 3% DMSO, and 1 μM of a pair of oligonucleotides: the sense primer that anneals within the gene of interest (5S, 16S, or 23S), and the antisense primer E4 DNA. Following separation on 3% agarose gels, PCR products were excised, purified, and sequenced after cloning using Zero Blunt TOPO PCR cloning kit (Invitrogen).

### cRACE

Purified rRNA fragments extracted from the agarose gel (S4 Fig) were circularized with 20 units T4 RNA ligase (Thermo Scientific), 1X RNA ligase buffer containing ATP, 15% DMSO, and 20 units of RNase Inhibitor in a 20-μl final reaction for 30 min at 37°C. After addition of 2 pmol of an RT primer, the reaction was adjusted to a final volume of 150 μl by adding milliQ water (RNase-free). Subsequently, the circularized RNAs were extracted with 1 volume of phenol-chloroform-isoamyl alcohol (P:C:I) in PLG tubes. The aqueous phase was mixed with 3 volumes of a mixture of ethanol and sodium acetate at pH 5, ratio 29:1, to precipitate the RNAs. Circularized RNA (0.25 μg) was converted to cDNA using an RT primer specific for each gene encoding the rRNAs and the Superscript III reverse transcriptase as described above (primer extension). The reverse transcripts were PCR amplified using PHUSION DNA polymerase (Finnzymes) and appropriate primers. The products were separated on a 3% agarose gel, purified, and then sequenced after cloning using the Zero Blunt TOPO PCR cloning kit (Invitrogen).

### RNA stability experiments

*E*. *coli* strains were grown on LB at 37°C to an OD_600_ of 0.4, and then rifampicin was added to a final concentration of 500 μg/ml to block new RNA synthesis. Incubation was continued at 37°C and aliquots were withdrawn at different time points (for example, 0, 1, 2, 4, 8, 16, and 32 min) after rifampicin addition, mixed with 0.2 volume of stop solution (5% phenol, 95% ethanol v/v), and directly snap frozen in liquid nitrogen. After thawing on ice and pelleting the cells, total RNA was extracted using TRIzol reagent. RNA levels were measured for the different time points after rifampicin treatment, by primer extension, and the relative half-lives of the 5S* RNAs were calculated. The quantities at different time points are plotted in a semi-logarithmic plot on Microsoft Excel, after normalization by defining the 0 time point (before rifampicin treatment) as having 100% RNA, using exponential fitting. The obtained decay curves appeared linear. The regressions curve function equation was used to determine the relative RNA half-lives.

### Purification of rRNA from polyacrylamide or agarose gels

DNase I-digested total RNA (5 to 10 μg) was resuspended with the RNA loading buffer (95% formamide, 0.1% xylene cyanol, 0.1% bromophenol blue, 10 mM EDTA), denatured for 5 min at 95°C, were separated by electrophoresis on 10% polyacrylamide gel in denaturing condition (7 M urea) in 1X TBE buffer (10X TBE: 890 mM Tris base, 890 mM boric acid, 20 mM EDTA) for 5 h 15 at 300 V. To purify 16S and 23S degradation fragments, RNA from sucrose fractions were separated on a 1% agarose gel in 1X TBE buffer for 3 h 30 at 50 V. After staining with SYBR Safe stain (Invitrogen) and visualization on ChemiDoc imager (Bio-Rad), the bands corresponding to 5S, 5S*, and the different degradation fragments of 16S and 23S were cut and extracted from the gel in 0.3 ml RNA elution buffer (0.1 M sodium acetate (pH 6.5), 0.1% SDS, and 10 mM EDTA (pH 8)) and incubated with agitation ON at 6 to 10°C. After centrifugation at 14,000 rpm for 15 min at 4°C, RNA was purified using Bio-Spin P30 columns (Bio-Rad). The RNAs were precipitated with 1 volume of absolute ethanol, eluted with 30 μl of milliQ water (RNase-free), and then stored at −20°C.

### Preparation of proteins from sucrose gradient fractions

Sucrose gradient fractions of 0.25 or 0.5 ml were collected, and proteins were precipitated by the addition of trichloroacetic acid (TCA_SIGMA) to a final concentration of 18%. The samples were mixed by inversion and frozen at −20°C for 30 min. The proteins were pelleted by centrifugation at 16,000*g* for 30 min at 4°C, and the protein pellets were washed twice with 0.3 ml of cold acetone. Acetone was then removed by centrifugation at 16,000*g* for 15 min at 4°C. The proteins were resuspended in 20 μl of 20 mM Tris–HCl (pH 7.5) and then denatured by addition of 2X Laemmli loading buffer (Laemmli 4X (Bio-Rad): 277.8 mM Tris–HCl (pH 6.8), 44.4% glycerol, 4.4% LDS, 0.02% Bromophenol blue) containing 5% β-mercaptoethanol followed by a heating step at 95°C for 5 min. Protein obtained after TCA precipitation (10 μl) was separated by 4% to 12% polyacrylamide gradient gel electrophoresis (NuPAGE-Invitrogen) in 1X MES buffer (50 mM MES, 50 mM Tris Base, 0.1% SDS 1 mM EDTA (pH 7.3)) for 1 h 35 at 120 V. After electrophoresis, the proteins were transferred to a nitrocellulose membrane (Bio-Rad) using a TransBlot transfer device (Bio-Rad) with the following parameters: mode: heterogeneous molecular weights, 25 V, Time, 7 min, and then subjected to western blotting.

### Western blotting

The blots were treated with anti-L5, anti-S3, and anti-L18 polyclonal antibodies provided by Isabelle Iost (INSERM, Bordeaux), hybridized with the second α-sheep-HRP antibody for 1 h at room temperature. Signals were visualized using the ECL kit (Bio-Rad) on a ChemiDoc Imager (Bio-Rad) for chemiluminescence detection.

### Determination of RNase E cleavage site in vivo and in vitro

To identify the cleavage sites of RNase E, sequences flanking the identified cleavages sites were aligned and analyzed by MEME suite (Version 4. 9. 1) to generate a consensus motif (http://meme-suite.org/) [79] as described [51].

### Ribosomal particles analysis by mass spectrometry

For mass spectrometry analysis, proteins from the different ribosomal particles were prepared in triple biological replicates for each strain. Proteins were extracted from the ribosomal particles after separation on sucrose gradient by adding 18% of cold acetic acid. Protein pellets were resuspended in 25 μl of cold 20 mM Tris–HCl (pH 7.5). Protein samples were reduced for 30 min with shaking at 56°C in 2X protein loading buffer (80 mM Tris–HCl (pH 6.8), 4% SDS, 20% glycerol, 0.16% BBP, 49.2 mM DTT) and then alkylated in 66 mM iodoacetamide (SIGMA) for 30 min in the dark at room temperature. Equal volumes of the obtained samples were loaded onto 4% to 12% Bis-Tris Nu-PAGE gel (Thermo Fisher). For one-shot analysis of the entire mixture, no fractionation was performed, and the electrophoretic migration was stopped as soon as the protein sample migrated for 0.5 cm. The gel was briefly stained using then InstantBlue (Expedeon Protein Solutions) according to the manufacturer’s instructions. Each single slice containing the whole sample was excised and subjected to in-gel tryptic digestion using modified porcine trypsin (Promega, France) at 10 ng/μl as previously described [83]. The dried peptide extracts obtained were dissolved in 12 μl of 0.05% trifluoroacetic acid in 2% acetonitrile and analyzed by online nanoLC using an Ultimate 3000 RSLCnano LC system (Thermo Scientific Dionex) coupled to an LTQ Orbitrap Velos mass spectrometer (Thermo Scientific, Bremen, Germany) for data-dependent CID fragmentation experiments. Each peptide extract (5 μl) was loaded in 2 or 3 injection replicates onto 300 μm ID × 5 mm PepMap C18 precolumn (Thermo Fisher, Dionex) at 20 μl/min in 2% acetonitrile, 0.05% trifluoroacetic acid. After 5 min of desalting, peptides were online separated on a 75-μm ID × 50 cm C18 column (in-house packed with Reprosil C18-AQ Pur 3 μm resin, Dr. Maisch; Proxeon Biosystems, Odense, Denmark), equilibrated in 95% of buffer A (0.2% formic acid), with a gradient of 5% to 25% of buffer B (80% acetonitrile, 0.2% formic acid) for 80 min then 25% to 50% for 30 min at a flow rate of 300 nL/min. The LTQ Orbitrap Velos was operated in data-dependent acquisition mode with the XCalibur software (version 2.0 SR2, Thermo Fisher Scientific). The survey scan MS was performed in the Orbitrap on the 350 to 1,800 m/z mass range with the resolution set to a value of 60,000. The 20 most intense ions per survey scan were selected with an isolation width of 2 m/z for subsequent data-dependent CID fragmentation, and the resulting fragments were analyzed in the linear trap (LTQ). The normalized collision energy was set to 30%. To prevent repetitive selection of the same peptide, the dynamic exclusion duration was set to 60 s with a 10-ppm tolerance around the selected precursor and its isotopes. Monoisotopic precursor selection was turned on. For internal calibration, the ion at 445.120025 m/z was used as lock mass.

### Database search and label-free quantitative analysis

All raw MS files were processed with MaxQuant (v 1.6.1.0) for database search with the Andromeda search engine and for quantitative analysis. Data were searched against the UniProtKB/Swiss-Prot protein database released 2018_04 with *E*. *coli* (K12 strain) (5,979 sequences) supplemented with a list of frequently observed contaminant sequences provided in MaxQuant. Enzyme specificity was set to trypsin/P, and a maximum of 2 missed cleavages was allowed. Carbamidomethylation of cysteines was set as a fixed modification, whereas methionine oxidation was set as variable modification. The precursor mass tolerance was set to 20 ppm for the first search and 10 ppm for the main Andromeda database search, and the mass tolerance in MS/MS mode was set to 0.8 Da. The required minimum peptide length was 7 amino acids, and the minimum number of unique peptides was set to 1. Andromeda results were validated by the target-decoy approach using a reverse database, and the false discovery rates at the peptide-spectrum matches (PSMs) and the protein level were set to 1%. For label-free relative quantification of the samples, the match between runs option of MaxQuant was enabled with a time window of 2 min, to allow cross-assignment of MS features detected in the different runs after alignment of the runs with a time window of 20 min. Protein quantification was based on razor peptides. The minimum ratio count was set to 1 for label-free quantification calculation, and computation of the intensity-based absolute quantification (iBAQ) metric was also enabled.

To perform relative quantification between all identified proteins, we used the normalized “LFQ intensity” metric from the MaxQuant “proteinGroups.txt” output. Protein groups with negative identification scores were filtered, as well as proteins identified as contaminants. After log2-transformation of LFQ intensities, log-transformed protein intensities corresponding to different technical LC–MS replicate runs were averaged and missing values were replaced to a mean LFQ intensity value was computed from technical LC–MS replicate runs by a noise value randomly drawn using the Perseus software (version 1.5.3.0). For each pairwise comparison of protein content of the subparticles 20S and 40S with their parental 30S and 50S from *rne*ΔMTS and with the 30S and 50S from *rne*+, an unpaired two-tailed Student *t* test was performed based on the protein intensities. Proteins were considered significantly enriched when their absolute log2-transformed fold change was higher than 1 and their *p*-value lower than 0.05. To eliminate false-positive hits from quantitation of low intensity signals, 2 additional criteria were applied: Only the proteins identified with a total number of averaged PSM counts >4 and quantified in a minimum of 2 biological replicates, before missing value replacement, for at least one of the 2 compared conditions were selected. Volcano plots were drawn to visualize significant protein abundance variations between the compared ribosomal particles. They represent -log10 (*p*-value) according to the log2 ratio. The complete list of the identified and quantified proteins and analyzed according to this statistical procedure is described in S2 Table.

### In vitro cleavage assay

Expression and purification of RNase E(1–598) with a C-terminal HISx6 tag in BL21(DE3) was as described [6]. Briefly, cells were lysed and debris were removed by centrifugation at 10,000*g*, 4° C for 1 h. The cleared lysate was applied to an NTA-Ni column and eluted with an imidazole gradient. Peak fractions were dialyzed overnight in storage buffer (10 mM Tris HCl (pH 7.5) - 500 mM NaCl—50% glycerol—0.2% Genapol X-080–10 mM MgSO_4_−1 mM EDTA—1 mM TCEP - 1x Protease Inhibitor), flash frozen in liquid N_2_, and stored at −80° C. Concentration was determined by UV absorbance at 280 nm using a molar extinction coefficient calculated from the amino acid composition of the protein. Ribosomes were purchased from NEB (P0763S). Ribosomal RNA was prepared by extraction of ribosomes using a Direct-zol RNA purification kit.

The RNase E cleavage assay was performed in a total reaction volume of 25 μl in either high ionic strength buffer (70 mM Tris (pH 7.5), 100 mM KCl, 25 mM MgCl_2_, 10 mM DTT, 2 U RNase inhibitor) or low ionic strength buffer (70 mM Tris (pH 7.5), 10 mM DTT, 2 U RNase inhibitor). Ribosomes (0.22 μM), or a comparable amount of rRNA (16 μg), were incubated with 0.3 μM RNase E(1–598)-Hisx6 at 37°C for 0, 30, or 60 min at 37°C. Reactions were quenched with 75 μl of cold 10 mM EDTA and held on ice. RNA was extracted using Direct-zol RNA purification kit, eluted in 60 μl water (RNase-free), lyophilized, and suspended in 10 μl of RNA loading dye (95% formamide, 0.1% xylene cyanol, 0.1% bromophenol blue, 10 mM EDTA). The sample was incubated for 3 min at 95°C and then separated on a 1% agarose gel in 1x TBE for 195 min at 50 V. The gel was stained with SYBR Green stain (Invitrogen).

## Supporting information

S1 Data The numerical values underlying Fig 1B. (XLSX)

S2 Data Numerical values used to generate the graphs in Figs 1C, 1E and 3C. (XLSX)

S1 Raw Images Uncropped images of the northern blots, PAA gels, slot blots, and western blots used in this study. (PDF)

S1 Table cRACE data underlying Fig 5A and 5B. (XLSX)

S2 Table Mass spectrometry data underlying Fig 6. (XLSX)

S1 Fig Bioanalyzer analyses of total RNA preparations. Electrophoretograms of total RNA isolated from cultures grown in minimal glucose medium at fast (μ = 0.6 h^−1^) and slow (μ = 0.1 h^−1^) growth rates. RNA levels were measured by fluorescence (FU), and elution was expressed either as seconds (s) or size (nt). The level of RNA in the peak centered at 100 nt was quantified as the percentage of total RNA. Under both fast and slow growth conditions, there was an approximately 60% increase in the level of Low Molecular Weight (LMW) RNA in the *rne*ΔMTS strain, suggesting an accumulation of RNA degradation products.
(TIF)

S2 Fig Ribosomal RNA 5′ end mapping by primer extension. Equal volume of clarified cell lysates from *rne*^+^ (left) and *rne*ΔMTS (right) strains were fractionated by velocity sedimentation under condition that optimized separation of monosomes and polysomes. RNA from the sucrose gradient fractions was analyzed by primer extensions using [^32^P] end-labelled oligonucleotides specific to the 5′ ends of 5S, 23S, 17S, and 16S rRNA. After extension by reverse transcriptase, the products were separated by denaturing gel electrophoresis. The 5′ end of mature rRNA and that of the prominent precursors are indicated to the right of each panel. Note that in both panels, there is a band located between 5S and p5S rRNA, which is present in 50S, 70S, and polysome fractions, which correspond to the 5S+1 and 5S+2 species. These species are poorly resolved due to a compression artifact in the migration of the cDNA. Uncropped gels of S2 Fig can be found in S1 Raw Images.
(TIF)

S3 Fig Mapping of rRNA 5′ ends by RACE. RNA from sucrose gradient fractions (Fig 2A) was analyzed by linker ligation to the RNA 5′ end, PCR amplification, and DNA sequencing (5′ RACE). 5′ ends were aligned with the sequence of 16S or 23S rRNA from the *E*. *coli rrfB* operon. 5′ extensions are indicated in red. Ribosomal RNA processing sites are indicated by arrows: RNase III, blue; RNase E, green; RNase AM, pink. The number of times each sequence was detected is indicated on the right. (**A)** 5S rRNA, (**B)** 23S rRNA, and (**C)** 16S rRNA.
(TIF)

S4 Fig Mapping of 5S and 5S* 3′ ends by RACE. 5S and 5S* rRNA species were extracted from the gel shown in Fig 3A and subjected to 5′ and 3′ RACE analysis. Reference sequences are from the *E*. *coli rrfB* operon. 5′ and 3′ extensions are indicated in red; untemplated extension in blue. RNase E processing sites are indicated by the green arrow. The occurrence for each sequence is summarized on the right (3′ RACE) or left side (5′ RACE). (**A)** 5′ end analysis of 5S* rRNA. 5′ RACE on 5S and 5S* rRNAs from *rne*ΔMTS strain. For comparison, 5S rRNA from *rne*^+^ strain was processed in parallel. (**B)** 3′ end analysis of 5S* rRNA. 3′ RACE on 5S and 5S* rRNAs from *rne*ΔMTS strain. For comparison, 5S rRNA from *rne*^+^ strain was processed in parallel. (**C)** As in (**B)** except in the *ΔpcnB* strain background.
(TIF)

S5 Fig Mapping of rRNA 3′ ends by RACE. RNA from sucrose gradient fractions (Fig 2A) was analyzed by linker ligation to the RNA 3′ end, PCR amplification, and DNA sequencing (3′ RACE). 3′ ends were aligned with the sequence of 5S or 23S rRNA from the *E*. *coli rrfB* operon. 3′ extensions are indicated in red; untemplated A additions in blue. 5S rRNA (**A**) and 23S rRNA (**B**) from the 50S subunit (*rne*^*+*^) and 40S particle (*rneΔMTS*).
(TIF)

S6 Fig Analysis of rRNA fragments by cRACE. Markup of gels shown in Fig 4A showing bands that were excised for RNA extraction and cRACE analysis. After RNA circularization, cDNA copies corresponding to the junction of the 5′ and 3′ ends were gel purified and cloned into a plasmid vector. The 5′-3′ ends were then identified by sequencing the cloned cDNA fragments. (**A)** In vivo fragments. (**B)** In vitro fragments. Uncropped gels of S6A and S6B Fig can be found in S1 Raw Images.
(TIF)

## Abbreviations

BR-bodies: Bacterial Ribonucleoprotein-bodies
cRACE: circular Rapid Amplification of cDNA Ends
DTT: dithiothreitol
FC: fold-change
*frt*: FLP recognition target
iBAQ: intensity-based absolute quantification
imRNase E: inner-membrane-RNase E
LMW: Low Molecular Weight
MTS: Membrane-Targeting Sequence
ncRNase E: nucleo-cytoplasmic-RNase E
PSM: peptide-spectrum match
RACE: rapid amplification of cDNA ends
RhlB: RNA helicase B
RNase E: ribonuclease E
rpm: rotations per minute
r-proteins: ribosomal proteins
SLiM: Short Linear Motif
