# Supplementary material for: Attachment of the RNA degradosome to the bacterial inner cytoplasmic membrane prevents wasteful degradation of rRNA in ribosome assembly intermediates
Source: PLoS Biol. 2023 Jan 5;21(1):e3001942. doi: 10.1371/journal.pbio.3001942 (PMC9848016; doi:10.1371/journal.pbio.3001942)
Supplement: S1 Raw Images — (PDF) [file pbio.3001942.s003.pdf]

Fig 1D: Ribosomal RNA levels.

Lanes not included in figure 1D are marked as X

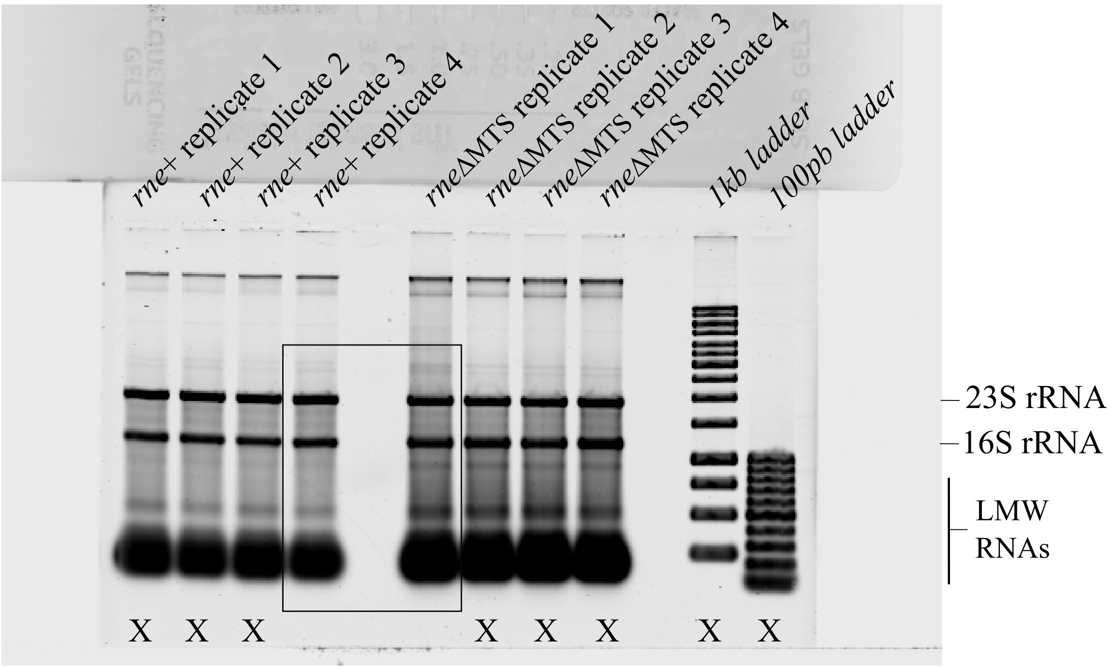

Image captured by Typhoon Trio- Amersham-Bioscience phosphoimager

Fig 2A: RNA content of ribosomal particles (Slot blots).

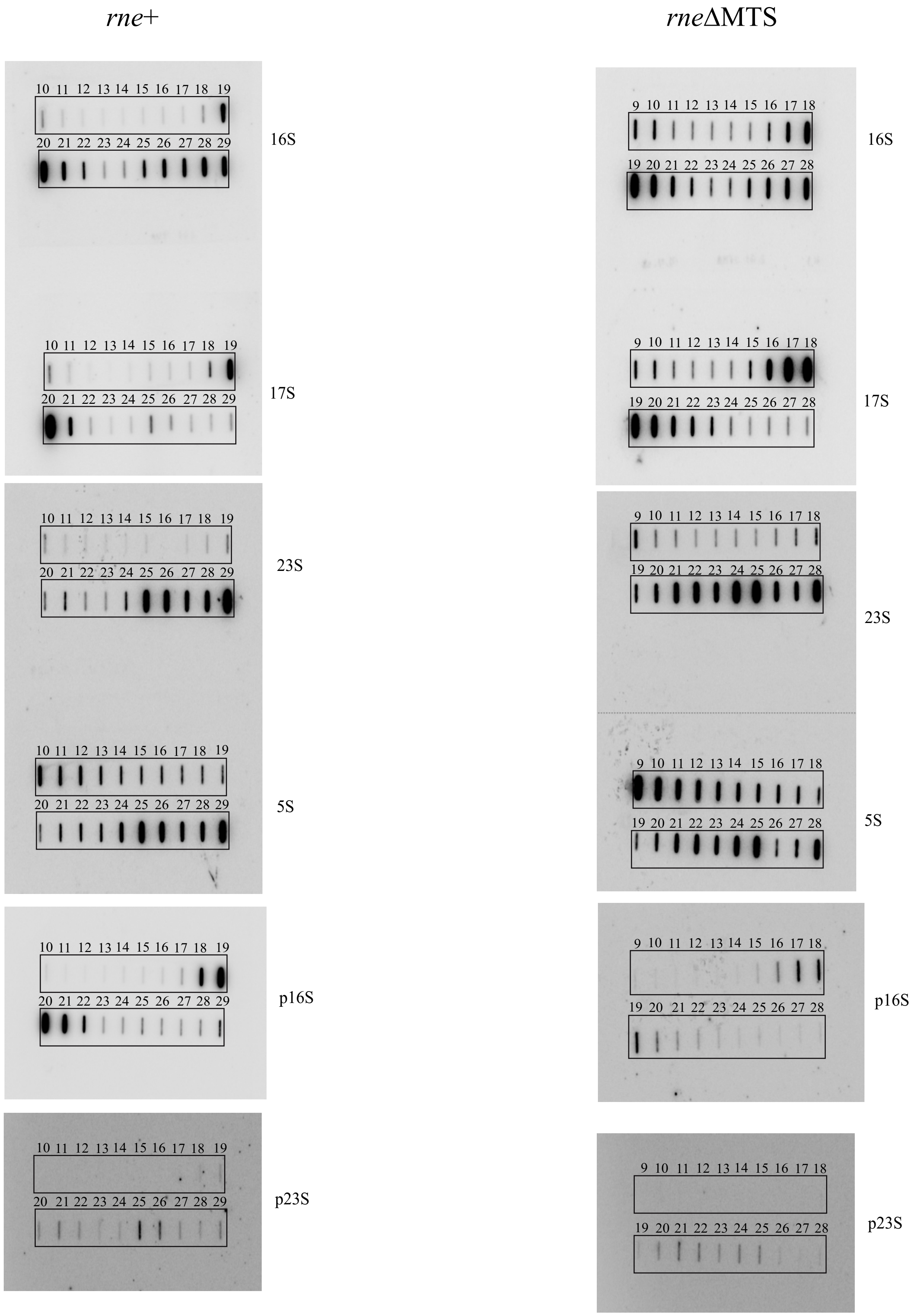

Images captured by Typhoon Trio- Amersham-Bioscience phosphoimager

Fig 2A_bottom: Protein content of ribosomal particles (western blots).

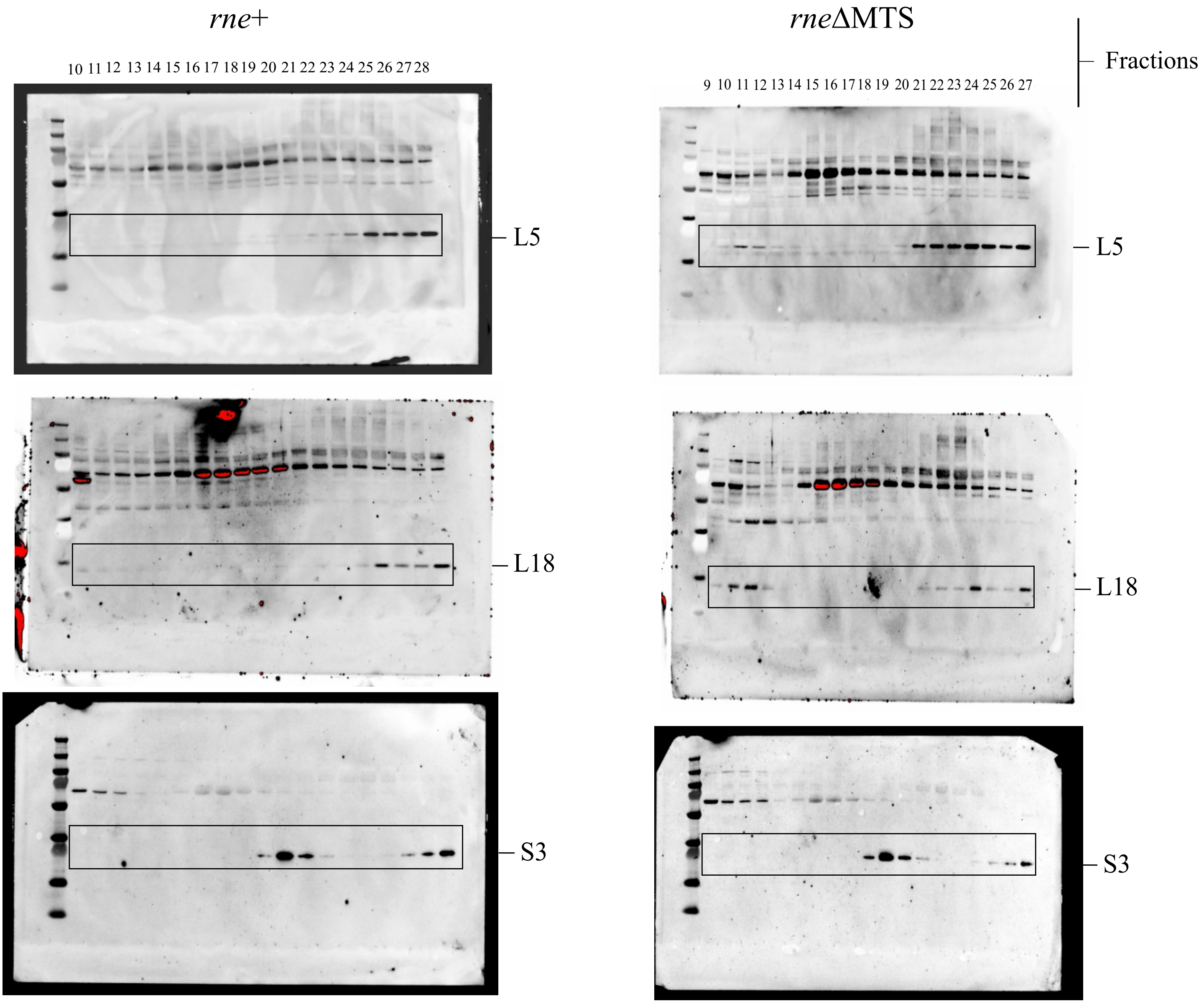

Images captured by ChemiDoc Imager (Biorad) for chemiluminescence detection

Fig 2B_part1: RNA content of ribosomal particles.

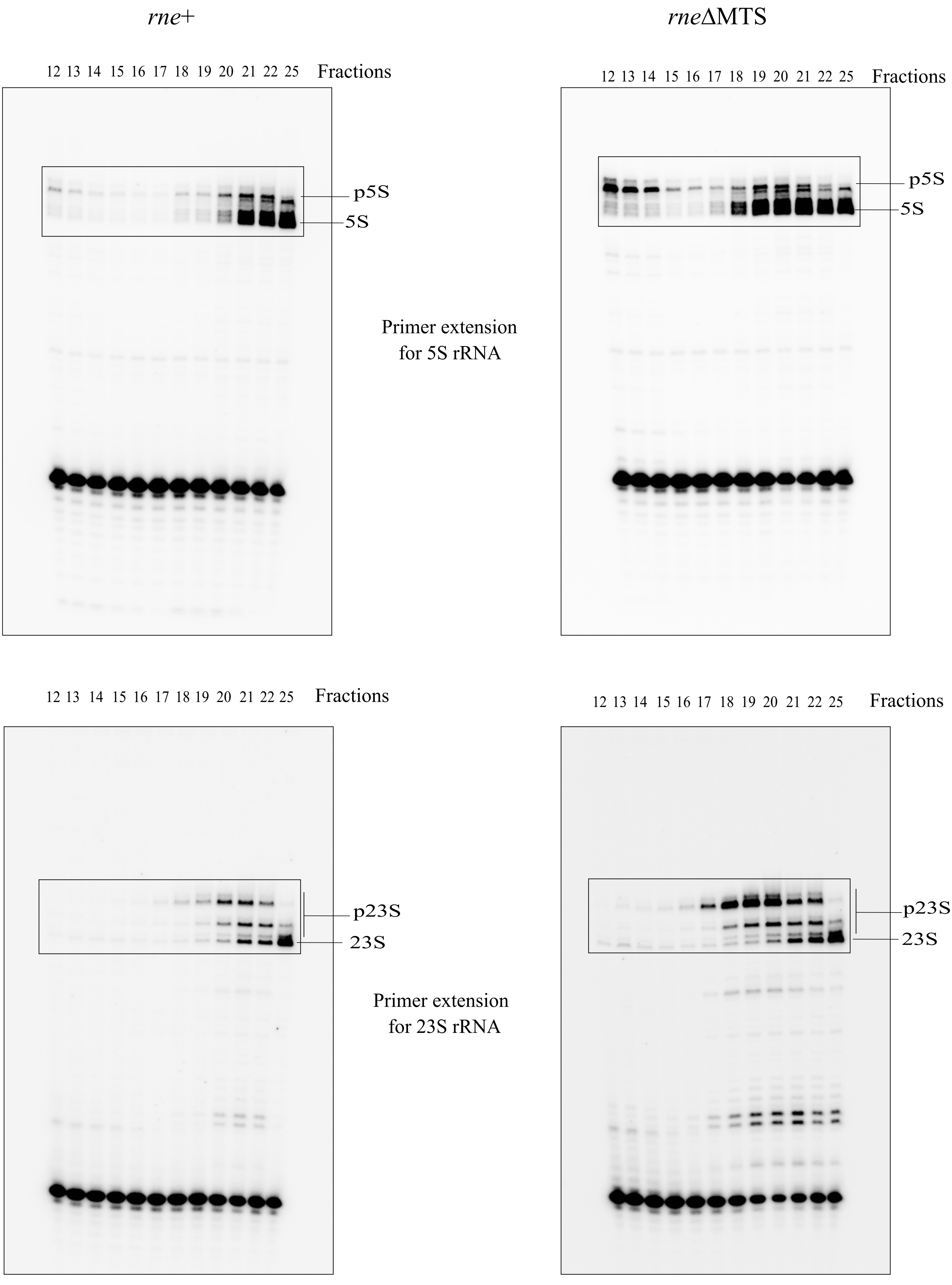

Images captured by Typhoon Trio- Amersham-Bioscience phosphoimager

Fig 2B_part2: RNA content of ribosomal particles.

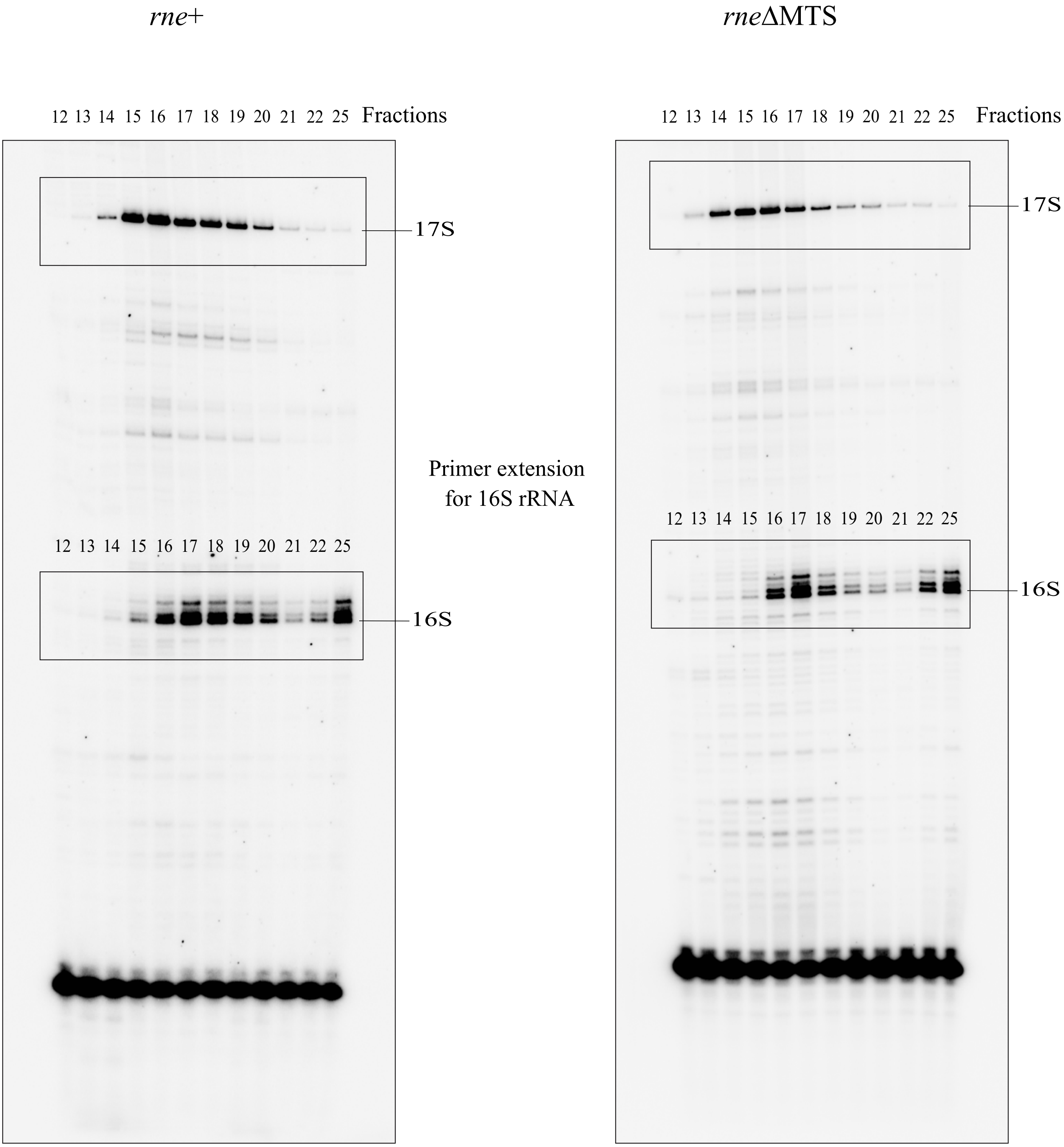

Images captured by Typhoon Trio- Amersham-Bioscience phosphoimager

Fig 3A.

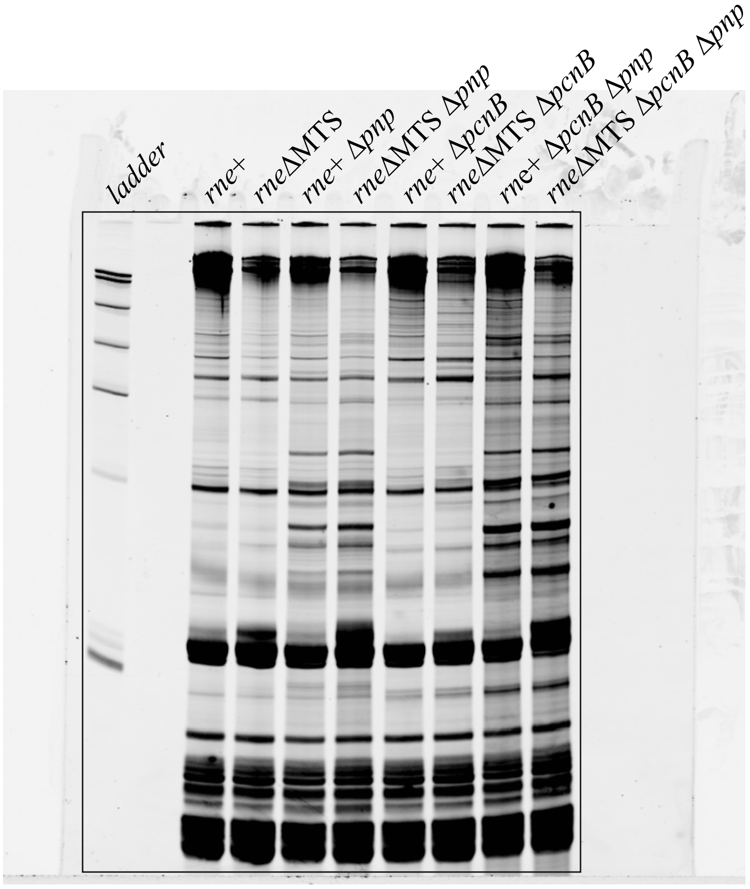

Fig 3B.

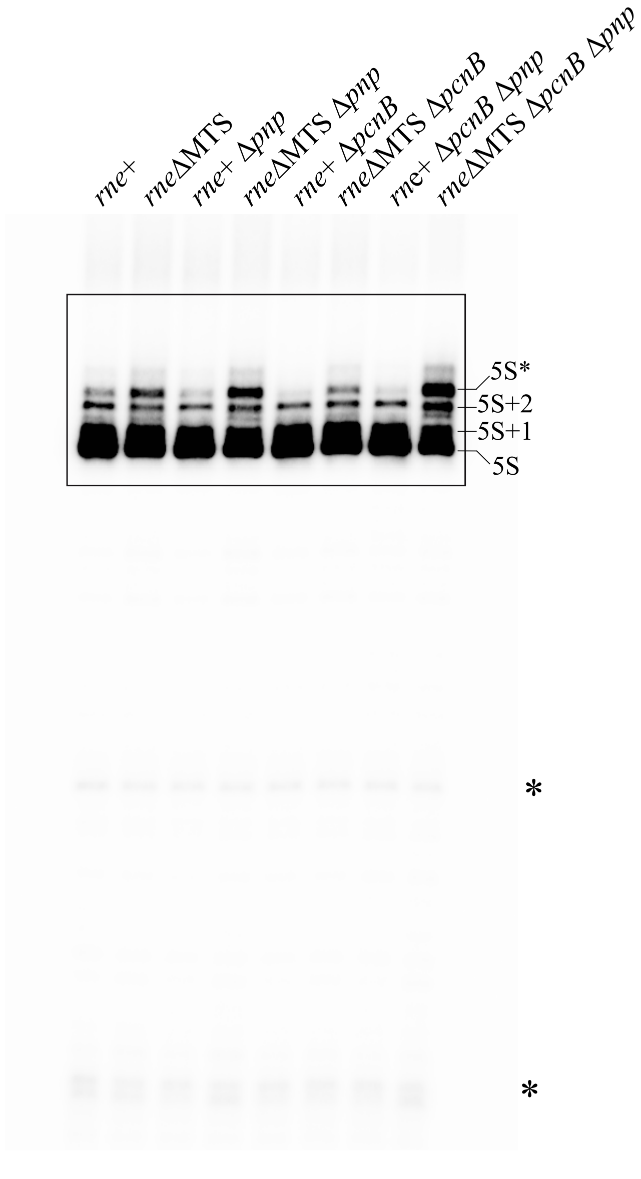

*Pausing of Reverse Transcriptase

Images captured by Typhoon Trio- Amersham-Bioscience phosphoimager

Fig 3C. part1

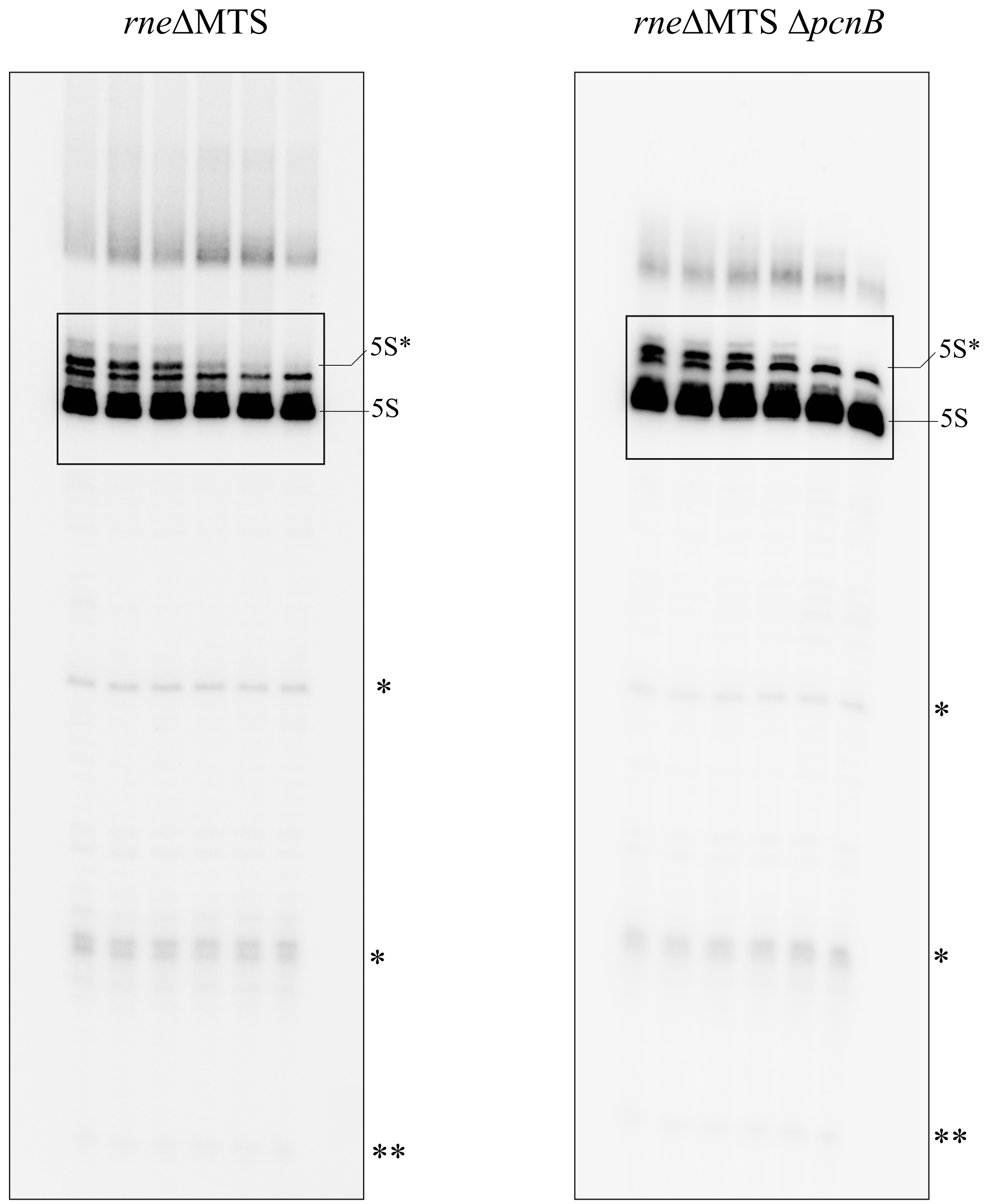

Images captured by Typhoon Trio- Amersham-Bioscience phosphoimager

*Pausing of Reverse Transcriptase

**Primer for reverse transcription (RT)

Fig 3C. part2

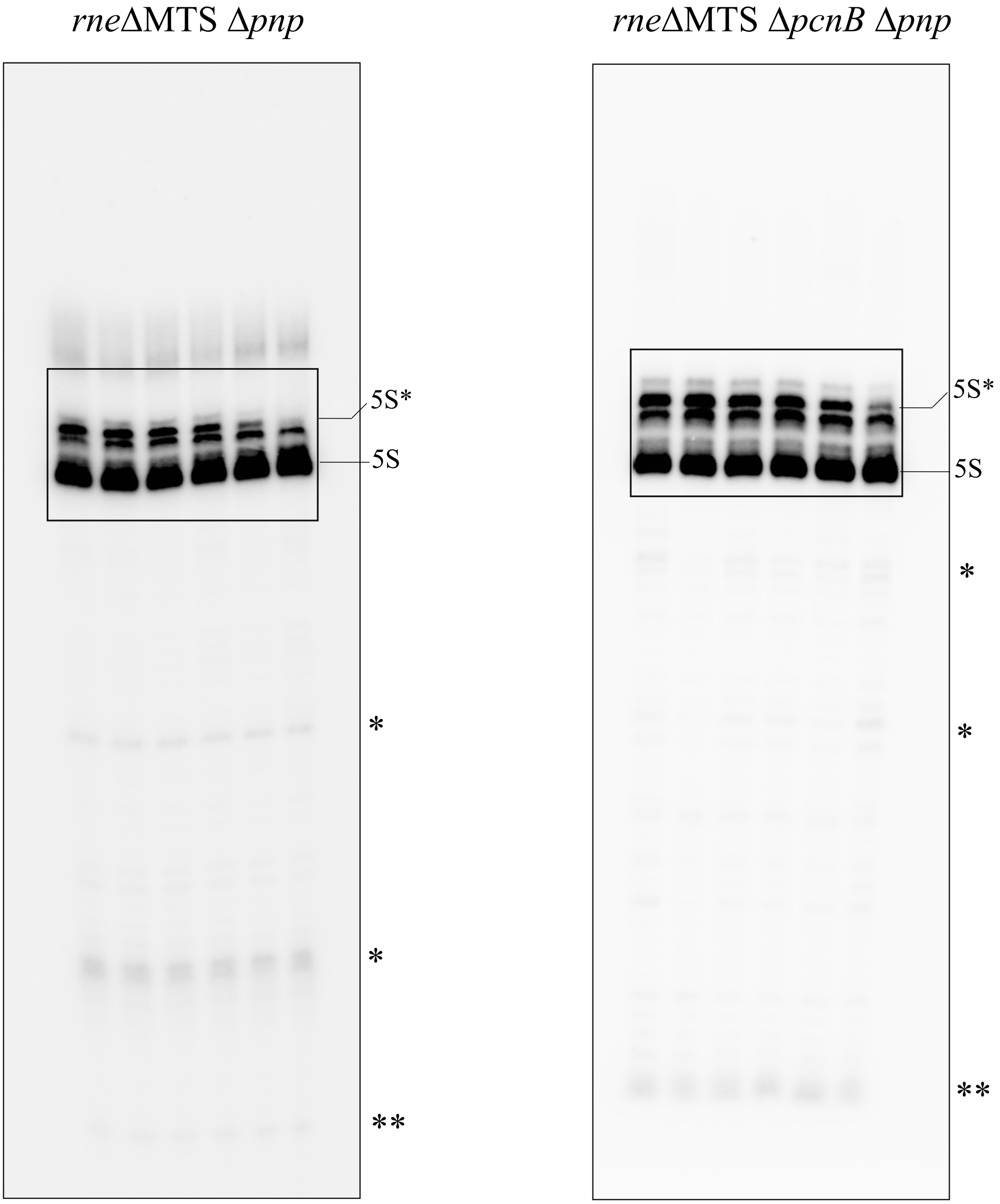

Images captured by Typhoon Trio- Amersham-Bioscience phosphoimager

*Pausing of Reverse Transcriptase

**Primer for reverse transcription (RT)

Fig 4A-B: Identification fo 16S and 23S rRNA fragments

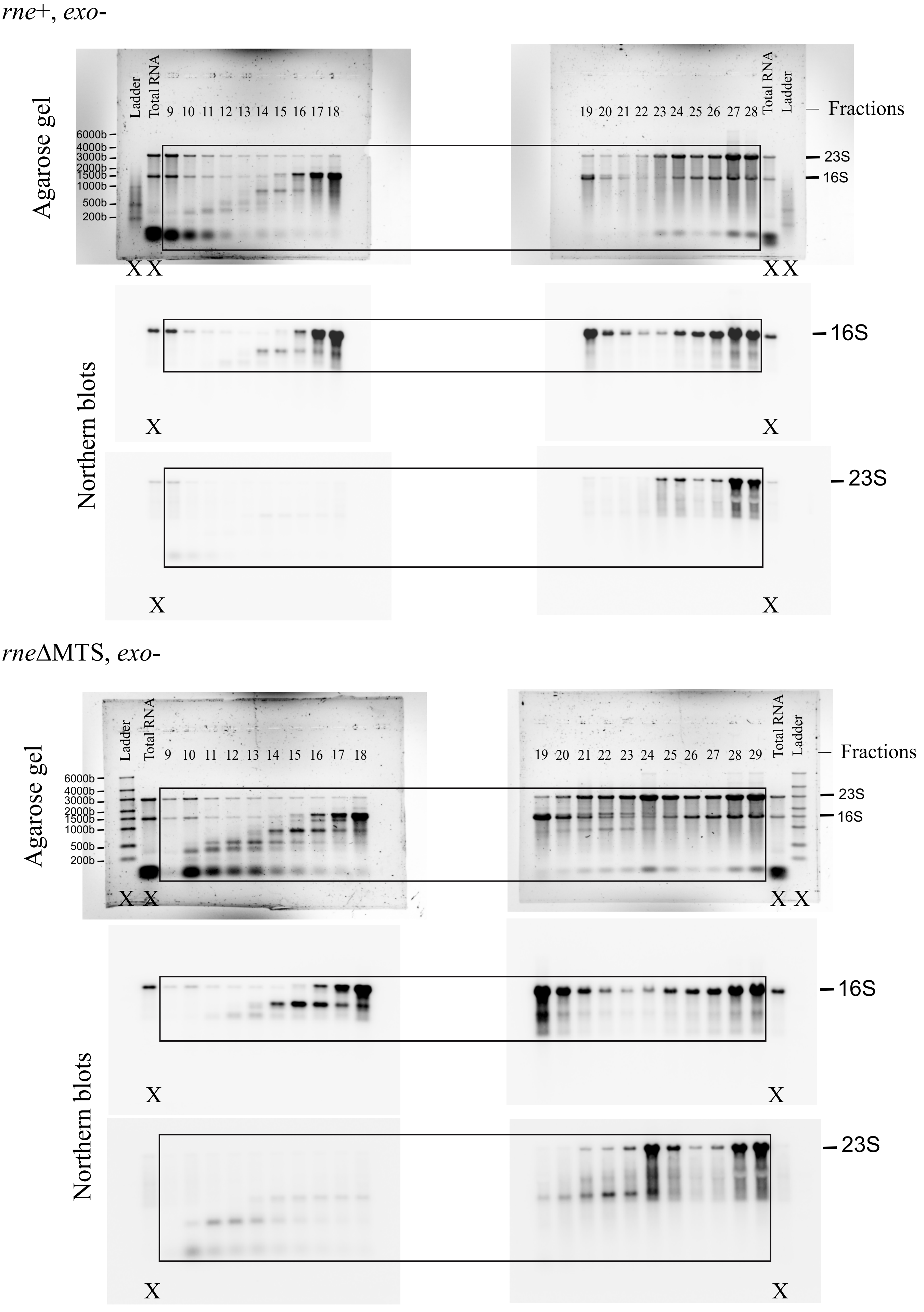

Lanes not included in figure 4 A-B are marked as X

Images captured by Typhoon Trio- Amersham-Bioscience phosphoimager

Fig 4C: Degradation of rRNA *in vitro*.

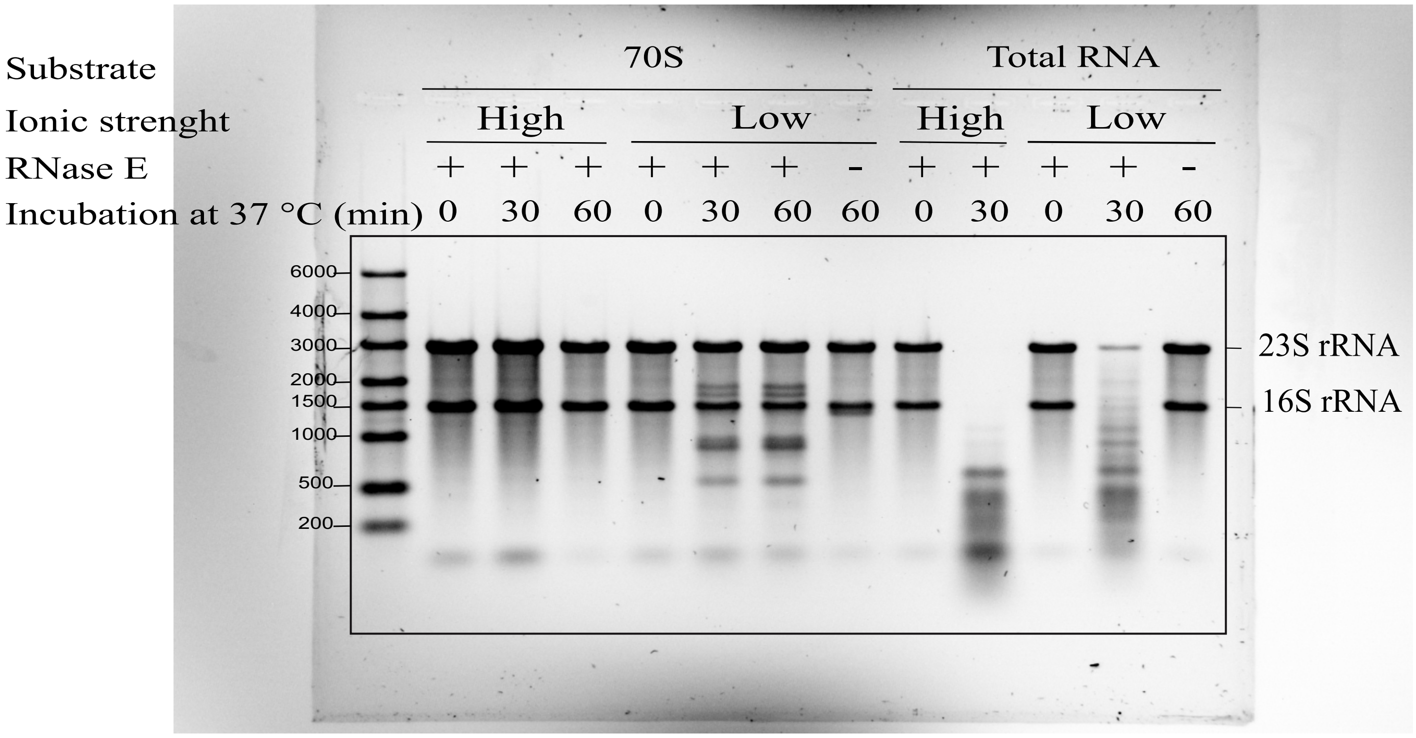

Image captured by Typhoon Trio- Amersham-Bioscience phosphoimager

Fig S2_part1: Ribosomal RNA 5' end mapping by primer extension.

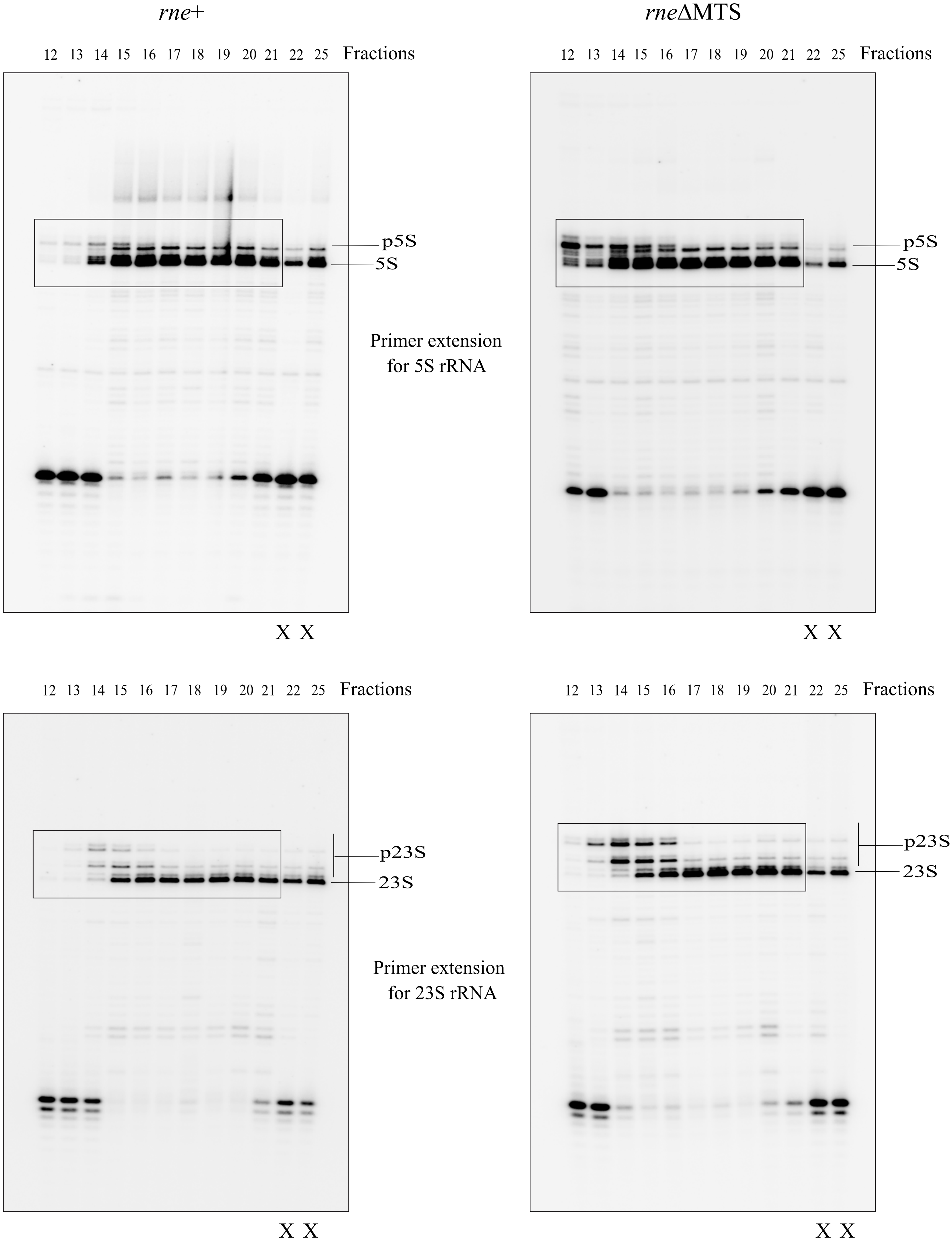

Lanes not included in figure S2 are marked as X

Images captured by Typhoon Trio- Amersham-Bioscience phosphoimager

Fig S2_part2: Ribosomal RNA 5' end mapping by primer extension.

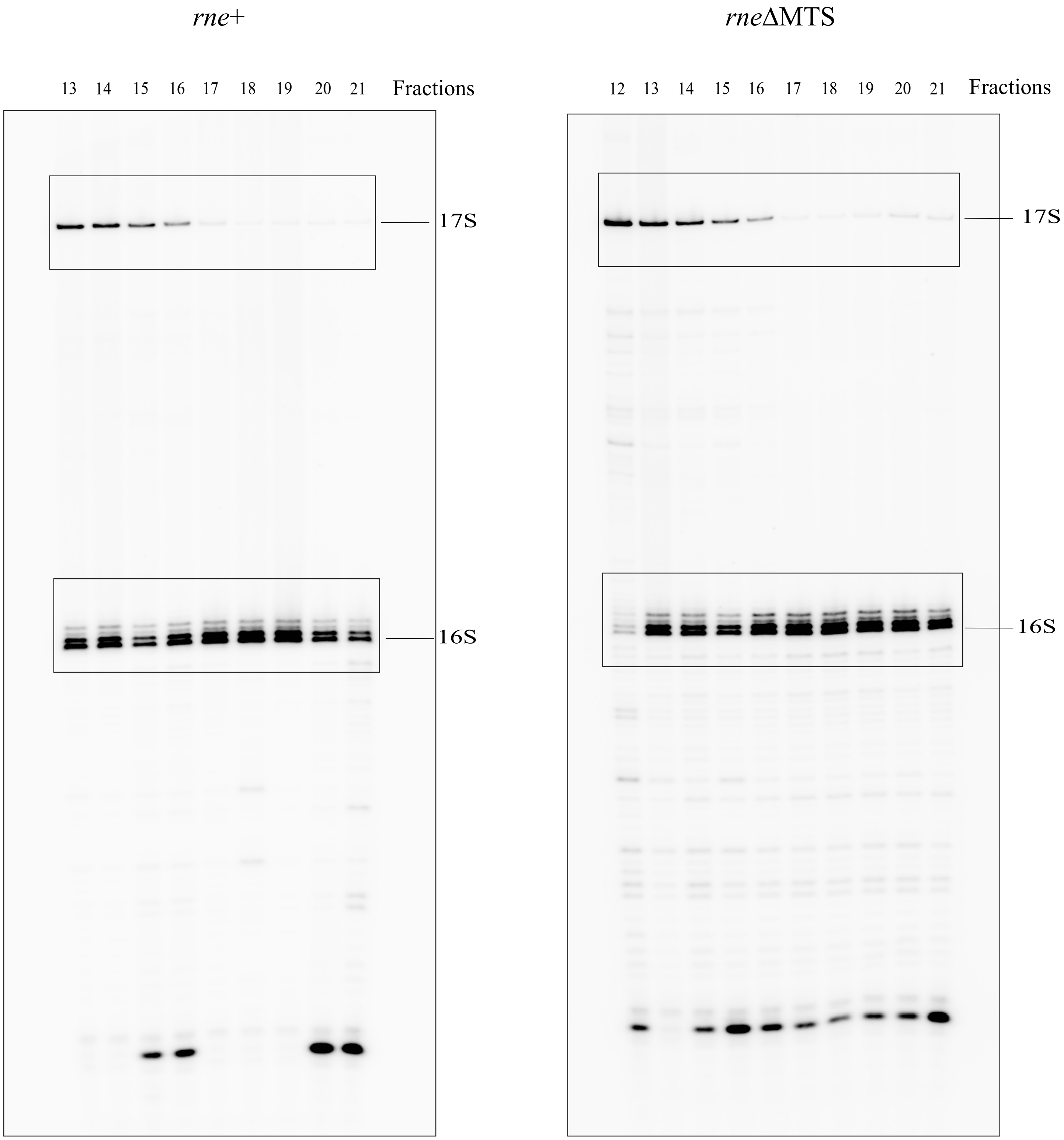

Primer extension for 16S rRNA

Images captured by Typhoon Trio- Amersham-Bioscience phosphoimager

Fig S6A: *In vivo*16S and 23S rRNA fragments extracted from the gel in Fig. 4A.

Lanes not included in figure S6A are marked as X

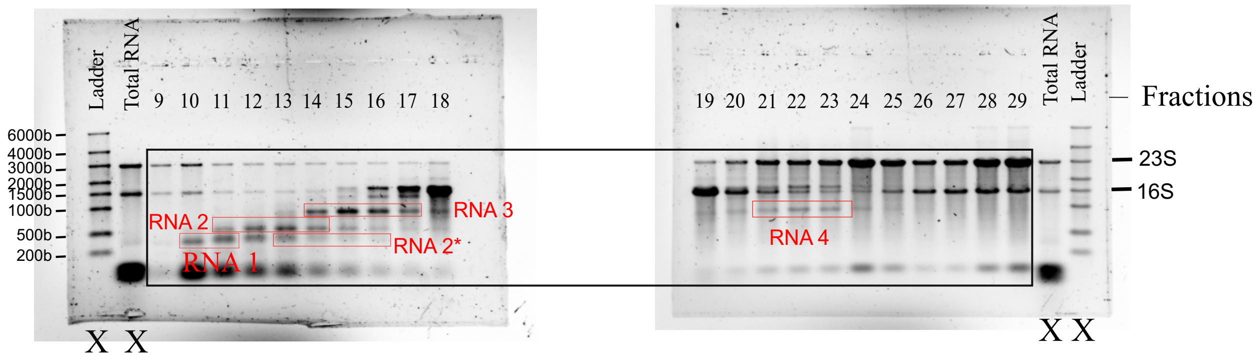

Image captured by Typhoon Trio- Amersham-Bioscience phosphoimager

Fig S6B: *in vitro* RNA fragments extracted from the gel in Fig 4C.

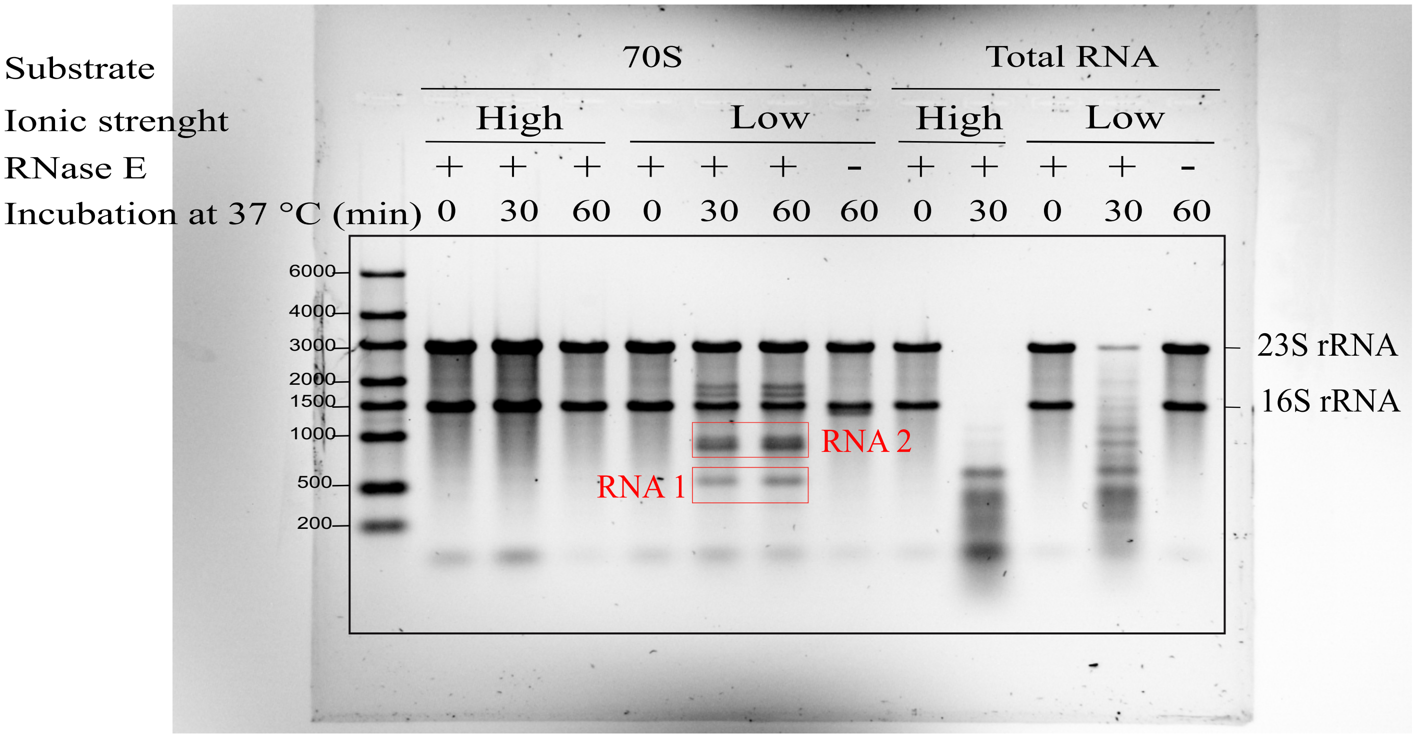

Image captured by Typhoon Trio- Amersham-Bioscience phosphoimager
