# Supplementary figures and images for: Attachment of the RNA degradosome to the bacterial inner cytoplasmic membrane prevents wasteful degradation of rRNA in ribosome assembly intermediates
Source: PLoS Biol. 2023 Jan 5;21(1):e3001942. doi: 10.1371/journal.pbio.3001942 (PMC9848016; doi:10.1371/journal.pbio.3001942)

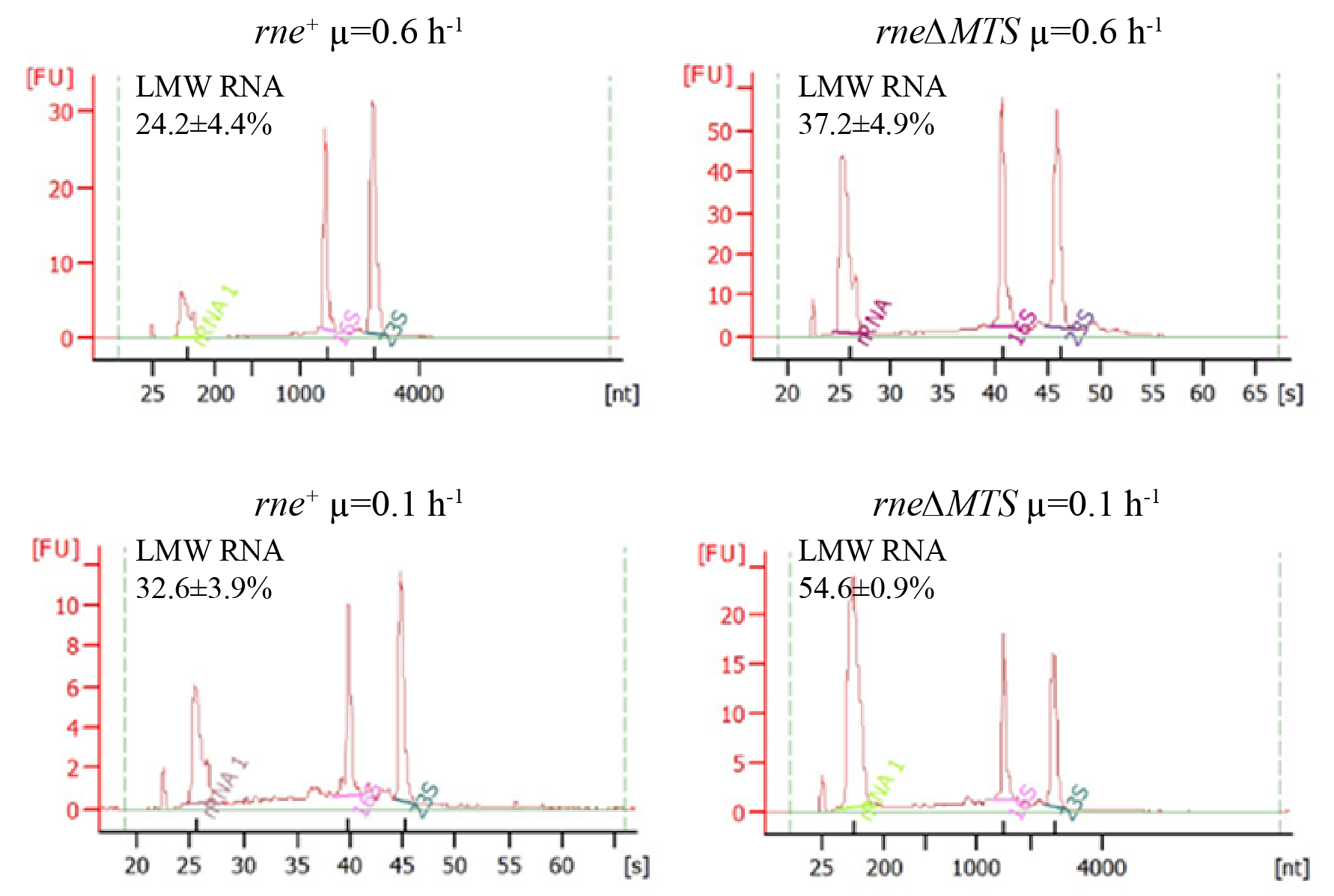

Supplement: S1 Fig — Electrophoretograms of total RNA isolated from cultures grown in minimal glucose medium at fast (μ = 0.6 h−1) and slow (μ = 0.1 h−1) growth rates. RNA levels were measured by fluorescence (FU), and elution was expressed either as seconds (s) or size (nt). The level of RNA in the peak centered at 100 nt was quantified as the percentage of total RNA. Under both fast and slow growth conditions, there was an approximately 60% increase in the level of Low Molecular Weight (LMW) RNA in the rneΔMTS strain, suggesting an accumulation of RNA degradation products. (TIF) [file pbio.3001942.s006.tif]

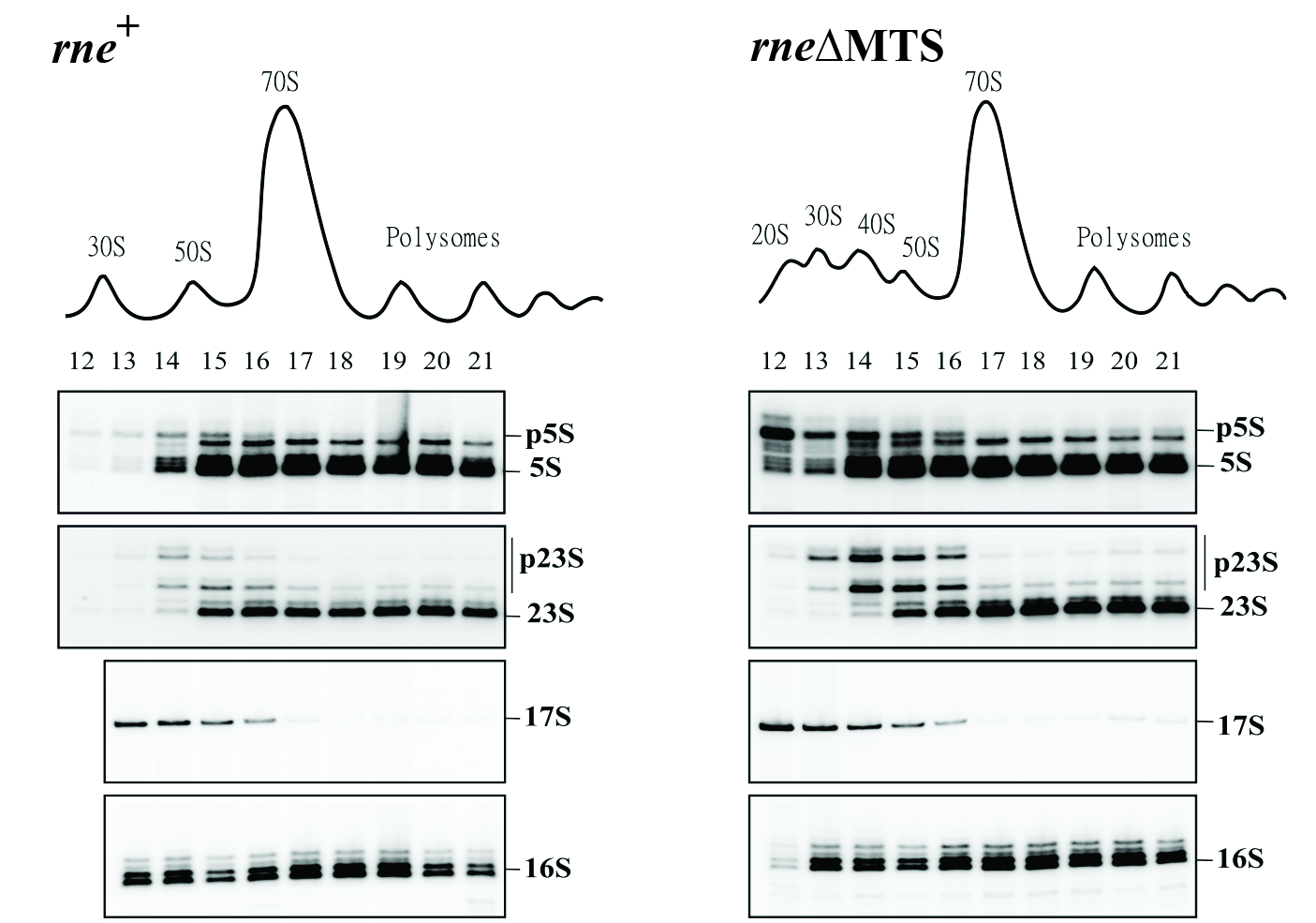

Supplement: S2 Fig — Equal volume of clarified cell lysates from rne+ (left) and rneΔMTS (right) strains were fractionated by velocity sedimentation under condition that optimized separation of monosomes and polysomes. RNA from the sucrose gradient fractions was analyzed by primer extensions using [32P] end-labelled oligonucleotides specific to the 5′ ends of 5S, 23S, 17S, and 16S rRNA. After extension by reverse transcriptase, the products were separated by denaturing gel electrophoresis. The 5′ end of mature rRNA and that of the prominent precursors are indicated to the right of each panel. Note that in both panels, there is a band located between 5S and p5S rRNA, which is present in 50S, 70S, and polysome fractions, which correspond to the 5S+1 and 5S+2 species. These species are poorly resolved due to a compression artifact in the migration of the cDNA. Uncropped gels of S2 Fig can be found in S1 Raw Images. (TIF) [file pbio.3001942.s007.tif]

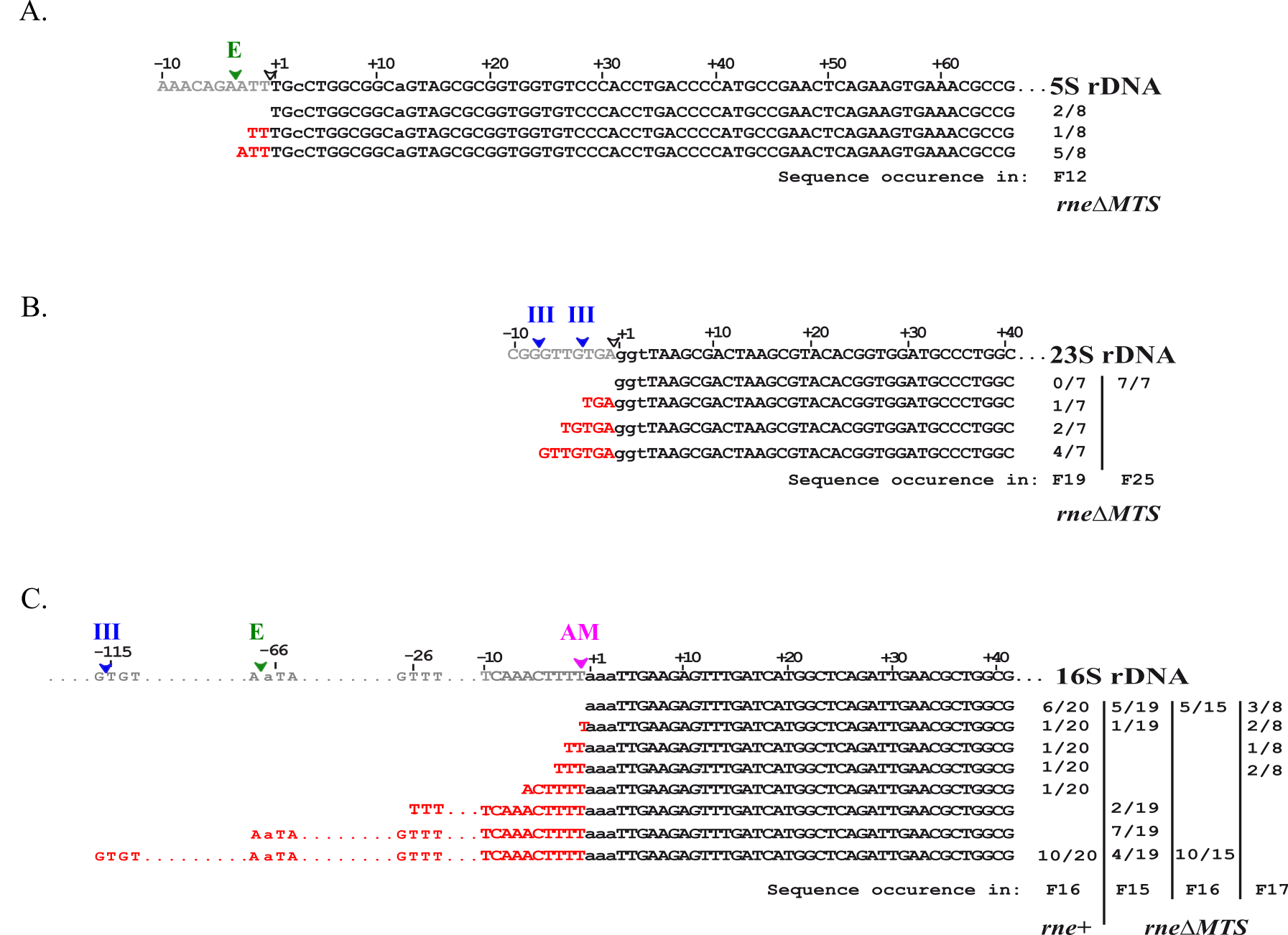

Supplement: S3 Fig — RNA from sucrose gradient fractions (Fig 2A) was analyzed by linker ligation to the RNA 5′ end, PCR amplification, and DNA sequencing (5′ RACE). 5′ ends were aligned with the sequence of 16S or 23S rRNA from the E. coli rrfB operon. 5′ extensions are indicated in red. Ribosomal RNA processing sites are indicated by arrows: RNase III, blue; RNase E, green; RNase AM, pink. The number of times each sequence was detected is indicated on the right. (A) 5S rRNA, (B) 23S rRNA, and (C) 16S rRNA. (TIF) [file pbio.3001942.s008.tif]

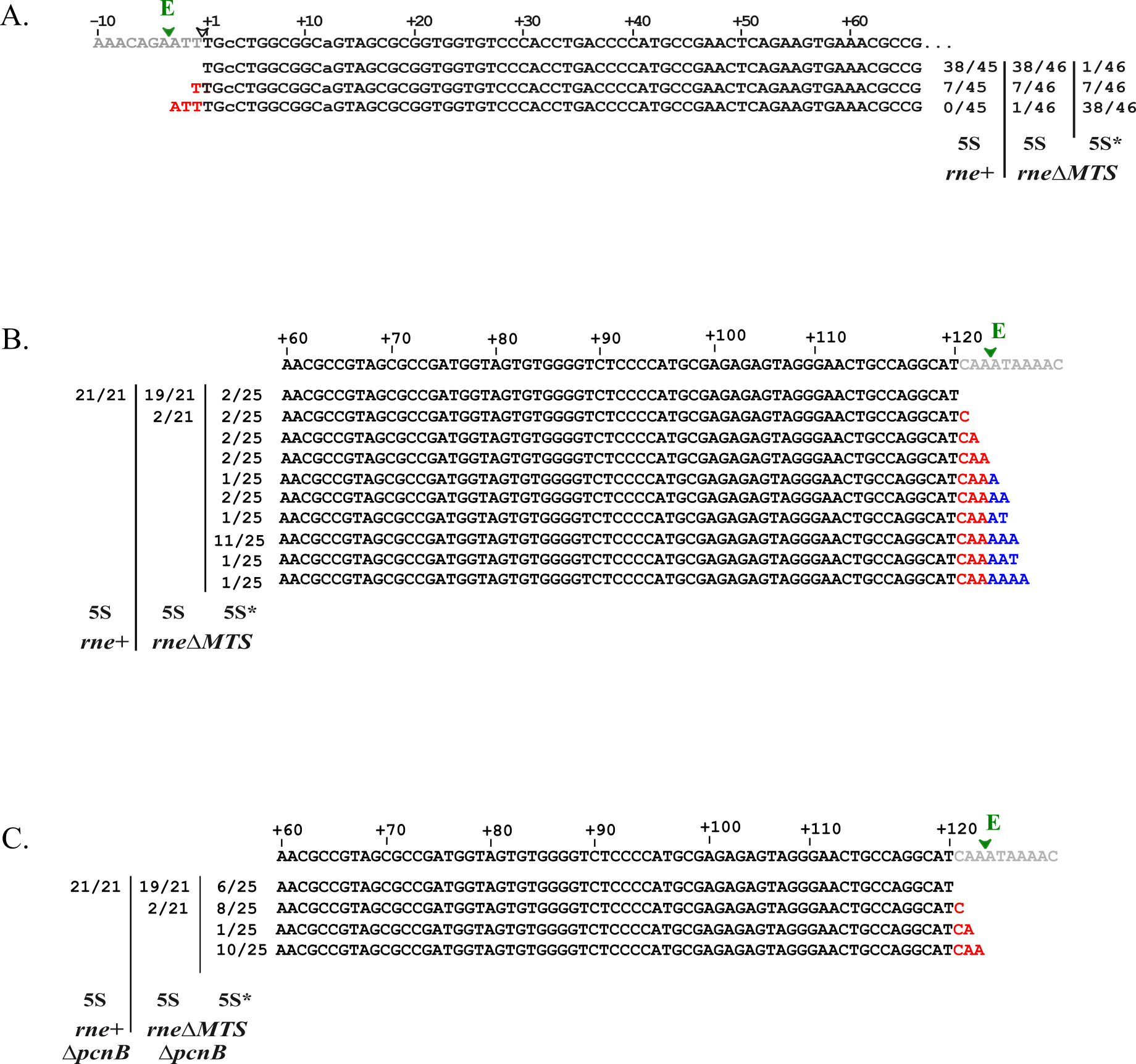

Supplement: S4 Fig — 5S and 5S* rRNA species were extracted from the gel shown in Fig 3A and subjected to 5′ and 3′ RACE analysis. Reference sequences are from the E. coli rrfB operon. 5′ and 3′ extensions are indicated in red; untemplated extension in blue. RNase E processing sites are indicated by the green arrow. The occurrence for each sequence is summarized on the right (3′ RACE) or left side (5′ RACE). (A) 5′ end analysis of 5S* rRNA. 5′ RACE on 5S and 5S* rRNAs from rneΔMTS strain. For comparison, 5S rRNA from rne+ strain was processed in parallel. (B) 3′ end analysis of 5S* rRNA. 3′ RACE on 5S and 5S* rRNAs from rneΔMTS strain. For comparison, 5S rRNA from rne+ strain was processed in parallel. (C) As in (B) except in the ΔpcnB strain background. (TIF) [file pbio.3001942.s009.tif]

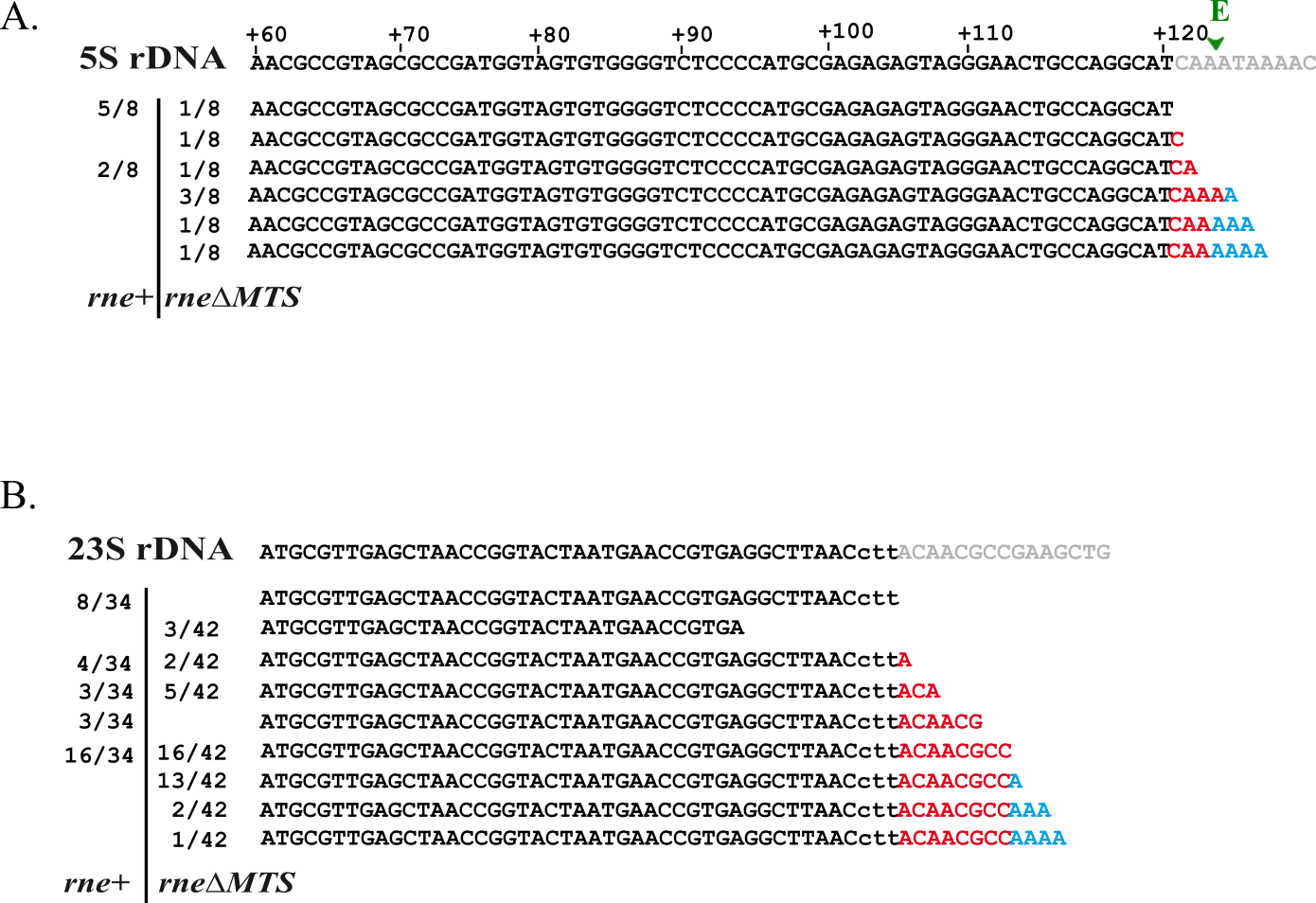

Supplement: S5 Fig — RNA from sucrose gradient fractions (Fig 2A) was analyzed by linker ligation to the RNA 3′ end, PCR amplification, and DNA sequencing (3′ RACE). 3′ ends were aligned with the sequence of 5S or 23S rRNA from the E. coli rrfB operon. 3′ extensions are indicated in red; untemplated A additions in blue. 5S rRNA (A) and 23S rRNA (B) from the 50S subunit (rne+) and 40S particle (rneΔMTS). (TIF) [file pbio.3001942.s010.tif]

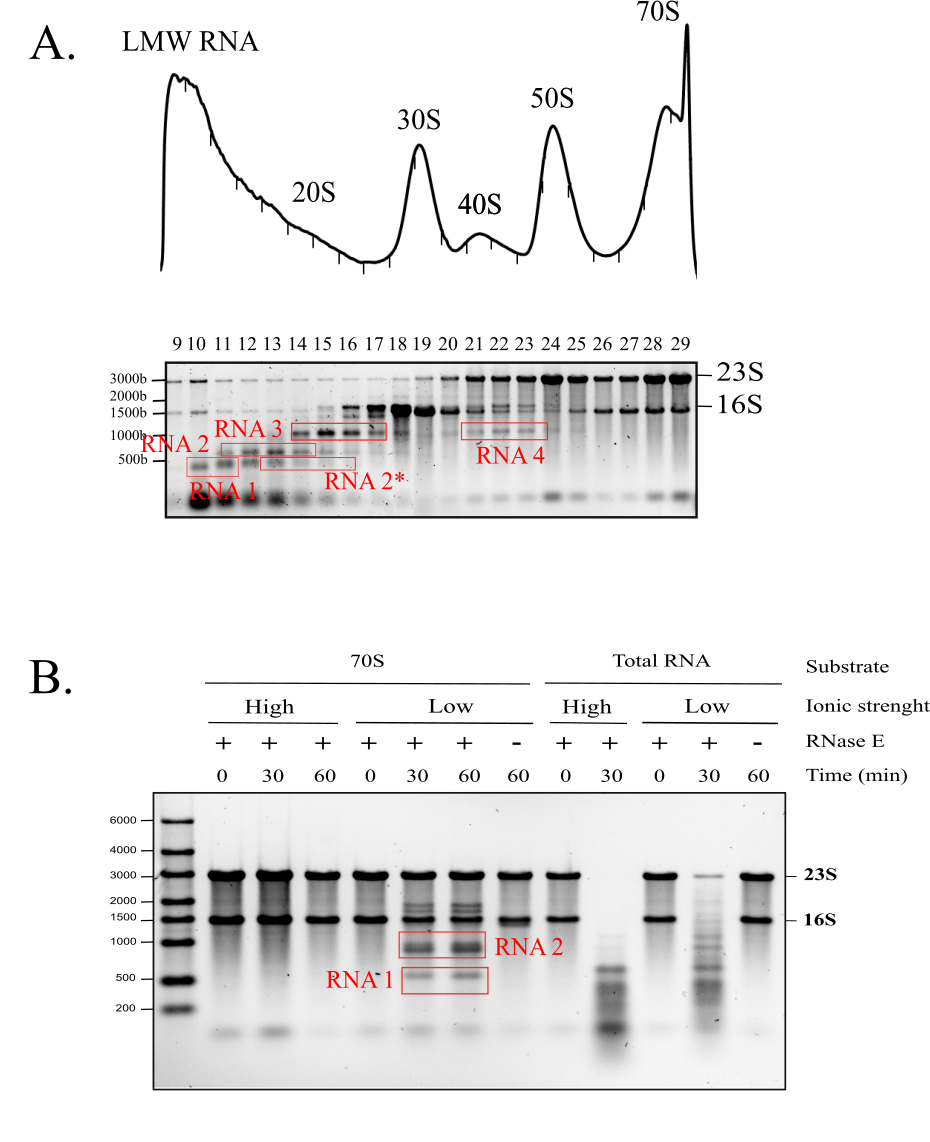

Supplement: S6 Fig — Markup of gels shown in Fig 4A showing bands that were excised for RNA extraction and cRACE analysis. After RNA circularization, cDNA copies corresponding to the junction of the 5′ and 3′ ends were gel purified and cloned into a plasmid vector. The 5′-3′ ends were then identified by sequencing the cloned cDNA fragments. (A) In vivo fragments. (B) In vitro fragments. Uncropped gels of S6A and S6B Fig can be found in S1 Raw Images. (TIF) [file pbio.3001942.s011.tif]
